# Off-Target Effects in Transgenic Mice: Characterization of Dopamine Transporter (DAT)-Cre Transgenic Mouse Lines Exposes Multiple Non-Dopaminergic Neuronal Clusters Available for Selective Targeting within Limbic Neurocircuitry

**DOI:** 10.1523/ENEURO.0198-19.2019

**Published:** 2019-10-08

**Authors:** Maria Papathanou, Sylvie Dumas, Hanna Pettersson, Lars Olson, Åsa Wallén-Mackenzie

**Affiliations:** 1 Department of Organismal Biology, Uppsala University, Uppsala 752 36, Sweden; 2 Oramacell, Paris 75006, France; 3 Department of Neuroscience, Biomedicum C4, Karolinska Institutet, Solna 17165, Sweden

**Keywords:** amygdala, Gad1, habenula, mammillary, septum, Vglut2

## Abstract

Transgenic mouse lines are instrumental in our attempt to understand brain function. Promoters driving transgenic expression of the gene encoding *Cre recombinase* are crucial to ensure selectivity in Cre-mediated targeting of floxed alleles using the Cre-Lox system. For the study of dopamine (DA) neurons, promoter sequences driving expression of the *Dopamine transporter* (*Dat*) gene are often implemented and several DAT-Cre transgenic mouse lines have been found to faithfully direct Cre activity to DA neurons. While evaluating an established DAT-Cre mouse line, reporter gene expression was unexpectedly identified in cell somas within the amygdala. To indiscriminately explore Cre activity in DAT-Cre transgenic lines, systematic whole-brain analysis of two DAT-Cre mouse lines was performed upon recombination with different types of floxed reporter alleles. Results were compared with data available from the Allen Institute for Brain Science. The results identified restricted DAT-Cre-driven reporter gene expression in cell clusters within several limbic areas, including amygdaloid and mammillary subnuclei, septum and habenula, areas classically associated with glutamatergic and GABAergic neurotransmission. While no *Dat* gene expression was detected, ample co-localization between DAT-Cre-driven reporter and markers for glutamatergic and GABAergic neurons was found. Upon viral injection of a fluorescent reporter into the amygdala and habenula, distinct projections from non-dopaminergic DAT-Cre neurons could be distinguished. The study demonstrates that DAT-Cre transgenic mice, beyond their usefulness in recombination of floxed alleles in DA neurons, could be implemented as tools to achieve selective targeting in restricted excitatory and inhibitory neuronal populations within the limbic neurocircuitry.

## Visual Abstract

**Figure:**
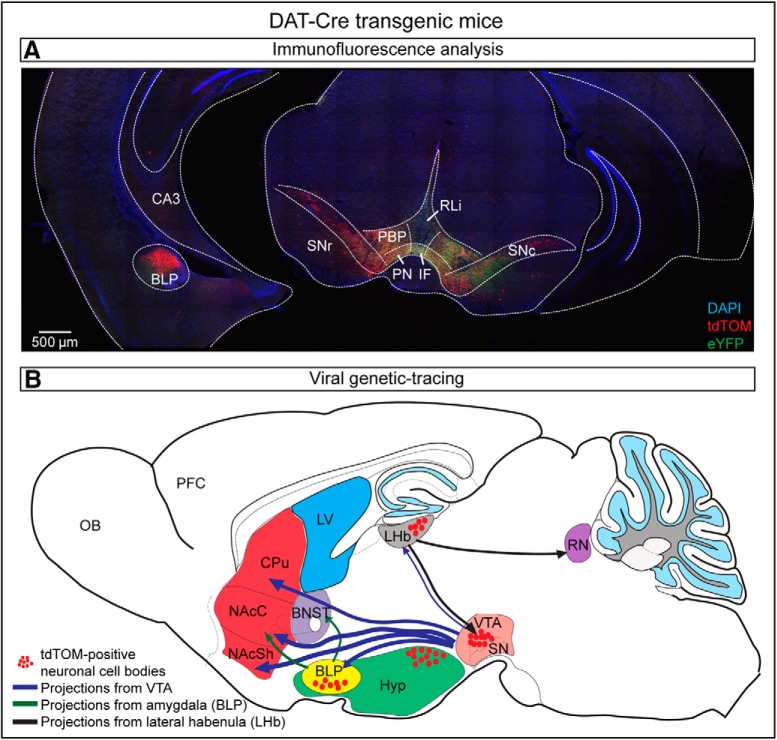

## Significance Statement

*DAT-Cre* transgenic mouse lines have been particularly useful in resolving the diverse functions of the brain’s dopamine (DA) systems. Here we report DAT-Cre-driven reporter gene expression in cell bodies of non-dopaminergic limbic brain areas, including the lateral septum, the amygdala and the lateral habenula (LHb). Co-labeling analysis identified that these DAT-Cre neurons were glutamatergic or GABAergic. Injection of viral-genetic constructs verified the activity of the DAT-Cre transgene in the adult brain, and also enabled identification of projection patterns. This study proposes a new angle by which available DAT-Cre transgenic mice can be implemented as tools for driving targeting to a restricted number of non-dopaminergic neurons of limbic neurocircuitry.

## Introduction

Transgenic mouse lines have been pivotal for advancing the knowledge of brain function, disentangling intricate neuronal circuits and enhancing insight into brain disorders (Gerfen et al., 2013). Midbrain dopamine (DA) neurons of the substantia nigra pars compacta (SNc; A9) and ventral tegmental area (VTA; A10) are essential for a wide range of functions, including voluntary movement, reward and motivation (Björklund and Dunnett, 2007; Ungless and Grace, 2012). Consequently, dysfunction of DA neurons is implicated in the pathophysiology of several neurologic and neuropsychiatric disorders, including Parkinson’s disease, schizophrenia, and addiction (Goldstein and Deutch, 1992; Volkow and Morales, 2015; Lüscher, 2016). Recent studies have identified a strong heterogeneity in the midbrain DA system, which is now recognized to consist of subtypes of DA neurons distinguished by distinct properties, including electrophysiological profile, molecular identity and ability for neurotransmitter co-release (for review, see Roeper, 2013; Lammel et al., 2014; Morales and Margolis, 2017). However, by definition, a dopaminergic neuron should express the gene encoding tyrosine hydroxylase (TH), the rate limiting enzyme for DA synthesis, while enzymes that convert DA into noradrenaline and adrenaline should be absent. In addition, most DA neurons express the gene encoding the DA transporter (DAT), which enables reuptake of extracellular DA into the cytosol (Jones et al., 1998; Smidt et al., 2003). Thus, despite increasing molecular knowledge of subtypes of DA neurons, the products of the *Th* and *Dat* genes are still considered the gold standards for identification of DA neuronal identity. Further, using the Cre-Lox system to achieve gene manipulation in transgenic animals, the promoters of the *Th* and *Dat* genes are frequently used as regulators of Cre recombinase to direct recombination of “floxed” (flanked by Lox sites) alleles to DA neurons.

A challenge with Cre-driven transgenics is the validation that expression of the Cre recombinase faithfully replicates the endogenous gene expression regulated by the selected promoter. Multiple factors account for how a transgene is expressed, including the genomic site of insertion, which if in an active locus may lead to ectopic expression of the transgene, i.e., transgenic expression in cells that normally do not express the endogenous gene. Further, developmentally active gene-regulatory sequences may lead to unanticipated Cre activity, which may cause gene-targeting of floxed alleles according to a temporally different schedule than intended. The specificity of TH-Cre and DAT-Cre transgenic mice has recently been debated (Lammel et al., 2015; Stuber et al., 2015). While TH-Cre mice have been shown to give rise to ectopic expression on recombination (Lindeberg et al., 2004; Savitt et al., 2005; Nordenankar et al., 2015a), several DAT-Cre transgenic lines have been shown to faithfully reproduce the gene expression pattern of the endogenous *Dat* gene in midbrain DA neurons (Zhuang et al., 2005; Bäckman et al., 2006; Ekstrand et al., 2007; Engblom et al., 2008). Further, viral injection of a Cre-dependent floxed reporter construct into the VTA of TH-Cre or DAT-Cre mice led to ectopic expression in the TH-Cre line while restricted to VTA TH-positive neurons in the DAT-Cre line (Lammel et al., 2015). DAT-Cre transgenic mouse lines thus provide a powerful tool to direct recombination of floxed alleles within DA neurons, both to achieve gene knock-out and to drive expression of floxed transgenes, e.g., optogenetic and reporter constructs. In addition to midbrain DA neurons, DAT-Cre transgenics has also proven useful for the study of neuroendocrine DA neurons of the hypothalamus (Soden et al., 2016; Stagkourakis et al., 2018b, 2019).

With a focus on DA populations in the context of DAT-Cre transgenes, non-DA neurons have been less explored. However, during the course of evaluation of a DAT-Cre transgenic mouse line using a floxed reporter line, reporter-positive cells were noticed within the basolateral amygdala, commonly associated with excitatory neurotransmission. To probe the possibility that ectopic DAT-Cre activity might be present in additional non-dopaminergic structures, a systematical analysis of two different DAT-Cre mouse lines was performed. The results were compared with yet one more DAT-Cre line published by the Allen Institute for Brain Science. The results identified DAT-Cre-driven reporter gene expression in defined clusters of the amygdala, lateral aspects of the septum and habenula, and in multiple additional areas in all three DAT-Cre lines. The majority of these unanticipated DAT-Cre neurons were identified as glutamatergic or GABAergic. Viral delivery of floxed reporters in the habenula and amygdala of adult DAT-Cre mice resulted in transfection of DAT-Cre neurons which enabled identification of distinct projections. The results show that DAT-Cre transgenic mice can be useful for the study of spatially restricted non-dopaminergic neurons within limbic neurocircuitry.

## Materials and Methods

### Animal housing

Animals of both sexes were housed on a standard 12 h sleep/wake cycle (7:00 A.M. lights on, 7:00 P.M. lights off). Mice were provided with food and water ad libitum and housed according to Swedish legislation (Animal Welfare Act SFS 1998:56) and European Union legislation (Convention ETS 123 and Directive 2010/63/EU). All experiments were conducted with permission from the local Animal Ethical Committee.

### Generation and genotyping of transgenic mice

Genotyping of transgenic mice was performed by PCR analysis (primer sequences, Table 1). Two different lines of transgenic mice expressing Cre *recombinase* under control of DAT promoter sequences were used: (1) DAT-Cre mice, in which Cre recombinase gene has been introduced into the endogenous DAT locus after an internal ribosomal entry site (IRES; so called DAT-IRES-Cre knock-in mice, abbreviated *DAT-Cre*; Ekstrand et al., 2007), and (2) *DAT-CreERT2* mice, in which an improved version of the Cre recombinase gene has been fused with a modified ligand-binding domain of the estrogen receptor and placed under control of a DAT promoter using BAC recombineering on pronuclear injection (Engblom et al., 2008). In the DAT-Cre line, Cre recombinase is hence expressed under control of the endogenous promoter, which has been shown to lead to Cre activity from an embryonal stage [around embryonal day (E)13; Kadkhodaei et al., 2009]. In the DAT-CreERT2 line, the transgenic construct has been randomly integrated into the genome. Only upon systemic treatment with tamoxifen will the Cre recombinase enter the nucleus and initiate recombination of floxed alleles, thus allowing temporal control of the recombination. Both DAT-Cre and DAT-CreERT2 mouse lines were bred to the floxed tdTomato reporter line (B6;129S6-*Gt (ROSA) 26Sor^tm14(CAG-tdTomato)Hze^*/J (The Jackson Laboratory), generating tdTom^DAT-Cre^ and tdTom^DAT-CreERT2^ mice. A subset of tdTom^DAT-Cre^ mice were injected with *AAV5-EF1a-DIO-eYFP* (see below). In addition, DAT-Cre mice were bred with the floxed *mCherryTRAP* reporter line (*Gt(ROSA)26Sor-mCherry-Rpl10a*; Hupe et al., 2014), generating mCherry TRAP^DAT-Cre^ mice. The use of two different reporters allowed visualization of different cellular structures: the tdTom reporter shows red fluorescence throughout the entire neuron, including fibers; the mCHERRY reporter shows red fluorescence in the cell soma only.

**Table 1.** PCR primer sequences used for genotyping of transgenic mice employed in the study

**Transgene**	**Direction**	**PCR primer sequence**
Dat-Cre	fw	5'-CACGACCAAGTGACAGCAAT-3'
Dat-Cre	rev	5'-AGAGACGGAAATCCATCGCT-3'
DAT-CreERT2	fw	5′-GGCTGGTGTGTCCATCCCTGAA-3′
DAT-CreERT2	rev	5′-GGTCAAATCCACAAAGCCTGGCA-3′
R26-Rpl10a	fw (mut)	5'-TACACCATCGTGGAACAGTAC-3'
R26-Rpl10a	rev (mut)	5'-GTAGTTCTTCAGGCTGATCTG-3'
R26-wt	fw (wt)	5'-GCGGATCACAAGCAATAATA-3'
R26-wt	rev (wt)	5'-TTTCTGGGAGTTCTCTGCTG-3'
tdTomato	fw (mut)	5′-CTGTTCCTGTACGGCATGG-3′
tdTomato	rev (mut)	5′-GGCATTAAAGCAGCGTATCC-3′
tdTomato	fw (wt)	5′-AAGGGAGCTGCAGTGGAGTA-3′
tdTomato	rev (wt)	5′-CCGAAAATCTGTGGGAAGTC-3′

### Tamoxifen administration

All experimental mice of the DAT-CreERT2 transgenic line were treated with tamoxifen to induce translocation of CreERT2 into the nucleus. Tamoxifen (Sigma, T-5648) was dissolved in sunflower oil and ethanol (9:1) to a final concentration of 20 mg/ml. Eight-week-old mice were injected intraperitoneally with 2 mg of tamoxifen once daily for five consecutive days. Animals were killed for further analysis one week after the last tamoxifen injection.

### Stereotaxic injection of AAV5-EF1a-DIO-eYFP

*AAV5-EF1a-DIO-eYFP* virus (UNC Gene Therapy) was used to express a floxed YFP reporter in adult mice on Cre-driven recombination. The *YFP* gene is inserted in reverse orientation relative to the 5' promoter and is flanked by loxP and lox2272 sites oriented in opposite direction. In the absence of Cre recombinase, YFP will not be expressed, while in the presence of Cre recombinase, the floxed YFP will be recombined/inversed and expressed. tdTom^DAT-Cre^ mice (more than eight weeks; >20 g) were anesthetized with isoflurane and stereotaxically injected with 300nl of AAV5-EF1a-DIO-eYFP (titer 6 × 10^12^ vg/ml; UNC Gene Therapy) at a flow-rate of 100 nl/min at the following coordinates from bregma: (amygdala) AP: –1.58, ML: ±2.5, and DV: –4.9; [lateral habenula (LHb)] AP: –1.7, ML: ±0.42, DV: –0.28; (VTA) AP: –3.45, ML: –0.2, and DV: –4.4 according to Paxinos and Franklin (2012). Three to four DAT-Cre mice were analyzed per stereotaxic coordinate. Topical analgesic, Marcaine (1.5 mg/kg; AstraZeneca) was applied during surgery and Caprofen (5 mg/kg; Norocarp) was given subcutaneously presurgery and postsurgery.

### Histologic analysis

#### Immunohistofluorescence analysis

Deeply anaesthetized mice were transcardially perfused with body-temperature PBS followed by ice-cold 4% formaldehyde. Brains were dissected and post-fixed for 1 h (for DAT antibody) or overnight (remaining antibodies). The brains were then cryo-protected with 30% sucrose and cut using a cryostat at 60-μm slice thickness. Free-floating sections were processed for immunofluorescence according to standard protocols. Sections were washed with PBS containing 0.1% Triton-X (PBS-T; 3 × 10 min) followed by 1 h of blocking (10% serum in 0.1% PBS-T) at room temperature (RT). Incubation of primary antibodies diluted in 0.1% PBS-T with 10% serum took place overnight at 4°C [rabbit TH 1:1000 #AB172, Millipore; chicken green fluorescent protein (GFP) 1:1000 #ab13970, Abcam (detects YFP); rabbit Calbindin (CALB1) 1:500 #AB1778, Millipore; rabbit Calretinin (CALB2) 1:500 #NBP1-88221, Novus Biologicals] or initiated for 2 h at RT and then overnight at 4°C (rat DAT 1:500 #MAB369, Millipore). Sections were subsequently washed in 0.1% PBS-T and incubated with the secondary antibodies in 0.1% PBS-T for 1 h at RT (donkey anti-rabbit Alexa Fluor 488 1:500; donkey anti-chicken Alexa Fluor 488 1:500; donkey anti-rat Alexa Fluor 488 1:500; donkey anti-rabbit Cy3 1:500) followed by washes in PBS and incubation of DAPI (1:5000, #D9542 Sigma-Aldrich) dissolved in water for 30 min at RT. Finally, sections were washed in PBS, mounted and coverslipped using ProLong antifade mounting medium (Invitrogen #P36970). tdTOM and mCHERRY were detected by their endogenous fluorescence without any additional detection enhancement by antibody. Three to four mice per genotype were analyzed. Images were captured using Mirax MIDI scanner, NanoZoomer 20.2-HT.0 or Zeiss Confocal (LSM 700, 20× magnification and z-stack with 1 or 0.5 zoom), and processed using PanoramicViewer (3DHISTECH), the Ndp2.view software (Hamammatsu) or Zen software (Zeiss) for maximum intensity projection.

#### Fluorescent and double-fluorescent in situ hybridization (sdFISH) analysis

The brains of anaesthetized mice were extracted and snap-frozen by rapidly immersing the tissue in ice-cold isopentane (–30°/–35°C). sdFISH was performed with riboprobes (Slc17a6 NM_080853.3 sequence: 2315–3244; Slc6a3 NM_012694.2 sequence 1015–1938; tdTom sequence: 84–653; Th NM_012740.3 sequence 456–1453, Gad1 NM_017007.1 sequence 174–1076) on 16-μm cryo-sections, which were briefly air-dried, fixed in 4% paraformaldehyde, acetylated in 0.25% acetic anhydride/100 mM triethanolamine (pH 8), and washed in PBS. Sections were hybridized for 18 h at 65°C in 100 μl of hybridization formamide-buffer containing 1 μg/ml digoxigenin (DIG) and 1 μg/ml fluorescein-labeled probes for detection of mRNA. Sections were washed at 65°C with SSC buffers (5× and 0.2×), followed by blocking with 20% heat-inactivated fetal bovine serum and 1% blocking solution (Roche #11096176001). Sections were next incubated with horseradish peroxidase (HRP)-conjugated anti-DIG antibody (Roche) at a dilution of 1:1000 in blocking solution. Signal detection was obtained with the TSA kit (PerkinElmer; NEL749A001KT) using biotin-tyramide at a dilution of 1:75 followed by incubation with NeutrAvidin Oregon green conjugate (Invitrogen) at 1:500. HRP activity was terminated by incubating the sections in 0.1 M glycine followed by a 3% H_2_O_2_ treatment. Fluorescein epitopes were then detected with HRP-conjugated anti-fluorescein antibody (Roche) at a dilution of 1:1000 in blocking solution and revealed with TSA kit (PerkinElmer; NEL744001KT) using Cy3-tyramide at a dilution of 1:150. All slides were scanned at 20× magnification on a NanoZoomer 2.0-HT. The Ndp2.view software (Hamamatsu) was employed for viewing the images. For each region of interest, a semi-quantitative histological analysis was performed by manual counting in a minimum of three sections per area per brain for the following probe combinations: TdTom/Dat, tdTom/Th, tdTom/Vglut2, tdTom/Gad1. A minimum of two mice per genotype and probe combination was analyzed.

## Results

### Two different DAT-Cre transgenic mouse lines show similar reporter gene expression in midbrain DA neurons of the VTA and SNc

To evaluate DAT-Cre transgenic mouse lines, we took advantage of two different DAT-Cre lines that we and others have previously used for the study of midbrain DA neurons (Engblom et al., 2008; Birgner et al., 2010; Alsiö et al., 2011; Kadkhodaei et al., 2013; Panman et al., 2014; Laguna et al., 2015; Wang et al., 2017; Papathanou et al., 2018; Bimpisidis et al., 2019): (1) a DAT-Cre knock-in line in which the Cre gene is expressed under control of the endogenous *Dat* promoter which leads to Cre recombinase activity already from embryogenesis (Ekstrand et al., 2007); (2) a tamoxifen-inducible DAT-CreERT2 line in which Cre recombinase translocates into the cell nucleus on systemic treatment with tamoxifen, a feature which allows Cre-dependent recombination at any age (Engblom et al., 2008). In the present study, tamoxifen was administered at the age of eight weeks to allow analysis of DAT-Cre-mediated recombination in adult mice.

**Figure 1.**
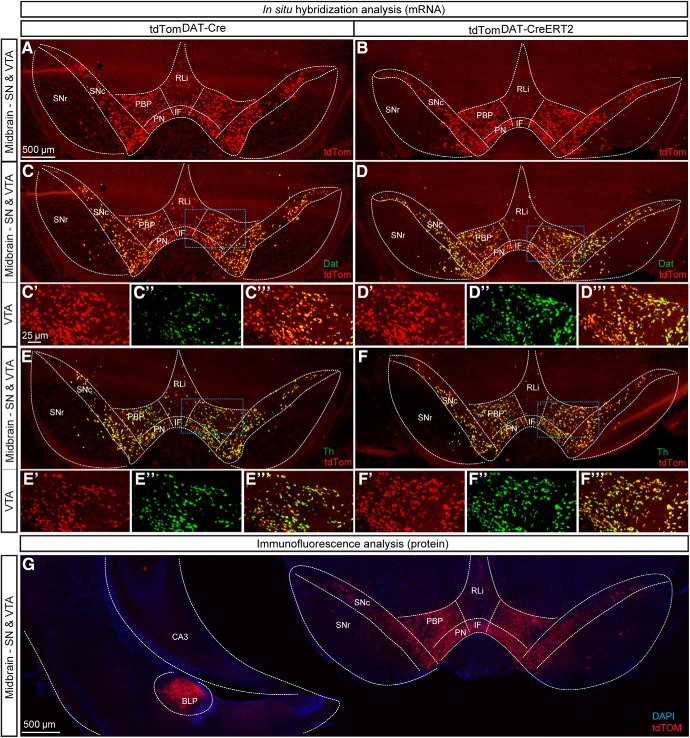
Histological analyses using double-*in situ* hybridization of tdTom and Dat or Th mRNA and immunofluorescence of tdTOM show selective recombination in the ventral midbrain using constitutive and tamoxifen-inducible DAT-Cre mouse lines and identifies ectopic tdTOM-positive neurons in the amygdala. tdTom mRNA in coronal sections of ventral midbrain of tdTom^DAT-Cre^ (***A***) and (***B***) tdTom^DAT-CreERT2^ mice 10 weeks of age. ***C***, ***D***, Double-FISH detecting tdTomato (red) and Dat (green) mRNA in the SNc and subregions of the VTA (IF, PBP, PN, and RLi) of the midbrain. Dat/tdTom mRNA overlap in the SNc and PBP shown in yellow (scale bar 500 μm). Higher magnification of insets of (***C’–C’’’***) tdTom^DAT-Cre^ and (***D’–D’’’***) tdTom^DAT-CreERT2^ mice (scale bar: 25 μm). ***E***, ***F***, Double-FISH for tdTom (red) and Th (green) mRNA in the SNc and VTA with co-localization shown in yellow (scale bar: 500 μm). Higher magnification of insets of (***E’–E’’’***) tdTom^DAT-Cre^ and (***F’–F’’’***) tdTom^DAT-CreERT2^ (scale bar: 25 μm). ***G***, Immunofluorescent coronal section of ventral midbrain showing tdTOM in the SN and VTA but also in BLP (scale bar: 500 μm). tdTom, tdTomato mRNA; tdTOM, tdTomato protein; CA3, CA3 region of hippocampus; BLP, Basolateral amygdaloid nucleus posterior part; Dat, Dopamine transporter; IF, Interfascicular nucleus; PBP, Parabrachial pigmented area; PN, Paranigral nuclei; RLi, Rostral linear nucleus; SNc, Substantia nigra *pars compacta*; SNr, Substantia nigra *pars reticulata*; tdTom, tdTomato mRNA; tdTOM, tdTomato protein; Th, Tyrosine hydroxylase; VTA: Ventral tegmental area; CA3, CA3 region of hippocampus.

By breeding the *DAT-Cre* line and the *DAT-CreERT2* line with the *tdTom* (Ai14) reporter mouse line, tdTom^DAT-Cre^ and *tdTom^DAT-CreERT2^* mice were produced in which reporter fluorescence visualizes any recombination activity achieved by the DAT-Cre-driven Cre recombinase. Midbrain DA neurons were first analyzed to validate expression of the reporter. Using sdFISH for fluorescent visualization of mRNA, tdTom mRNA was identified throughout the VTA and the SNc (Fig. 1*A*,*B*). Within the VTA subareas known as the parabrachial pigmented area (PBP), paranigral nucleus (PN), rostal linear nucleus (RLi), and interfascicular nucleus (IF), ample tdTom-positive cells were detected in both tdTom^DAT-Cre^ and tdTom^DAT-CreERT2^ mice (Fig. 1*A*,*B*). Apart from scattered tdTom-fluorescent cells in the SN pars reticulata (SNr), reporter gene expression was only found in the VTA and SNc of the ventral midbrain. Next, the spatial distribution of tdTom mRNA was compared with Dat and Th mRNAs by tdTom/Dat and tdTom/Th sdFISH co-localization analysis. tdTom and Dat mRNA were found throughout the VTA and SNc with the most substantial overlap between these mRNAs seen in the PBP of the VTA and in the SNc in both the tdTom^DAT-Cre^ (Fig. 1*C–C’*,*C’’’*) and tdTom^DAT-CreERT2^ (Fig. 1*D–D’*,*D’’’*) mice. For both Cre lines, more sparse co-localization was seen in the VTA subareas RLi and PN, in which the Dat mRNA signal was weaker. In contrast, a comparative analysis between tdTom and Th mRNAs showed that almost all tdTom neurons were Th-positive (Fig. 1*E–E’*,*E’’’*,*F–F’*,*F’’’*).

Next, direct fluorescence from the tdTOM reporter protein was addressed which confirmed the presence of tdTOM-protein-positive neurons in the VTA and SNc (Fig. 1*G*). At the same section level, tdTOM-positive cell bodies were also observed in the amygdala of the tdTom^DAT-Cre^ mice (Fig. 1*G*). By comparison with a brain atlas (Paxinos and Franklin, 2012), these tdTOM-positive cells appeared to represent a distinct cell cluster in the posterior subdivision of the basolateral amygdala (BLP). This observation motivated whole brain analysis on serial sections from both tdTom^DAT-Cre^ and tdTom^DAT-CreERT2^ transgenic mice.

### Clusters of tdTOM-positive cell bodies detected in several forebrain areas including the lateral septum, lateral habenula, premammillary nucleus of the hypothalamus and retromammillary nucleus, and distinct subareas of the amygdaloid complex

**Figure 2.**
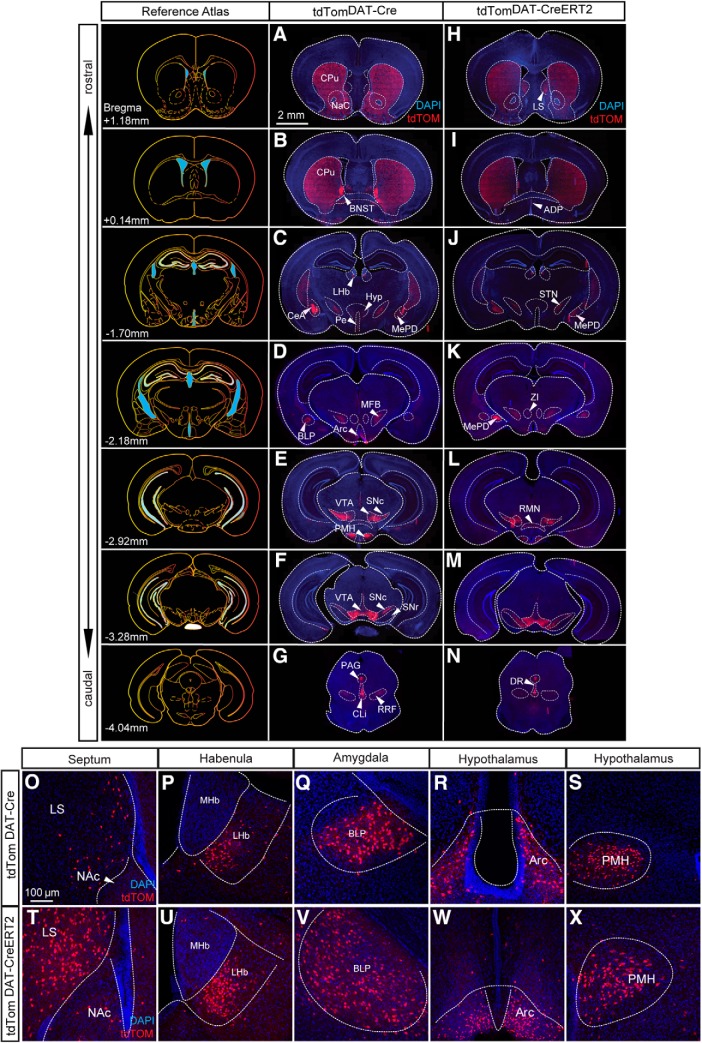
Immunohistofluorescent analysis of brains derived from tdTom^DAT-Cre^ and tdTom^DAT-CreERT2^ mice identifies tdTOM-positive cell bodies in the LS, LHb, and the amygdala. Series of coronal sections from the level of the striatum and NAc through to the raphe nucleus showing expression pattern of recombination in (***A–G***) tdTom^DAT-Cre^ and (***H–N***) tdTom^DAT-CreERT2^ mice (scale bar: 2 mm). Expression of tdTOM-positive neurons in the (***O***, ***T***) lateral septum, (***P***, ***U***) LHb, (***Q***, ***V***) BLP, (***R***, ***W***) arcuate nucleus, and (***S***, ***X***) the premammilary nucleus of the hypothalamus in tdTom^DAT-Cre^ and tdTom^DAT-CreERT2^ mice (scale bar: 100 μm). ADP, anterodorsal preoptic nucleus; Arc, arcuate nucleus; CPu, caudate putamen; DR, dorsal raphe; Hyp, hypothalamus; LS, lateral septum; MFB, medial forebrain bundle; MHb, medial habenula; PAG, periaquaductal gray; Pe, periventricular hypothalamic nucleus; STN, subthalamic nucleus; ZI, zona incerta; BLP, Basolateral amygdaloid nucleus posterior division; BNST, Bed nucleus of the stria terminalis; CeA, Central amygdala, CLi, Caudal linear nucleus; NAc, Nucleus accumbens; MePD, Medial amygdaloid nucleus posterodorsal part, and LHb, Lateral habenula, PMH, Premammillary nucleus; RMN, Retromammillary nucleus; RRF, Retrorubral field; SNc, Substantia nigra pars compacta; SNr, Substantia nigra pars reticulata; VTA: Ventral tegmental area.

Fluorescent microscopy of serial sections throughout the brain of tdTom^DAT-Cre^ and tdTom^DAT-CreERT2^ mice was performed to examine the presence tdTOM-positive neurons. In tdTom^DAT-Cre^ or tdTom^DAT-CreERT2^ mice lacking expression of Cre recombinase, no tdTOM-positive cells or fibers were detected in any brain area. However, in Cre-positive mice, fluorescence from the TdTOM protein was readily detected in brain areas known to contain DA neurons, including the SNc and VTA (all VTA subareas listed above as well as the caudal linear nucleus, CLi) as well as in the retrorubral field (RRF; A8), the periaqueductal gray (PAG), the glomerular layer of the olfactory bulb, the zona incerta (A13), the anterodorsal preoptic area (A14) and within the periventricular nucleus and the arcuate nucleus of the hypothalamus (A12) in both tdTom^DAT-Cre^ (Fig. 2*A–G*,*R*) and tdTom^DAT-CreERT2^ (Fig. 2*H–N*,*W*) mice. Further, projections of the midbrain and hypothalamic DA systems showed tdTOM fluorescence: The median forebrain bundle, the striatal complex as well as other areas innervated by projections from DA neurons regions were positive for tdTOM in both tdTom^DAT-Cre^ (Fig. 2*A–G*) and tdTom^DAT-CreERT2^ (Fig. 2*H–N*) mice.

In addition to DA-producing neurons, tdTOM-derived fluorescence was also detected in the soma of neurons commonly associated with excitatory and inhibitory neurotransmission: the lateral septum (Fig. 2*A*,*H*,*O*,*T*), the ventromedial aspect of the lateral habenula (LHb) (Fig. 2*C*,*J*,*P*,*U*), the basolateral amygdaloid nucleus posterior part (BLP) (Fig. 2*D*,*K*,*Q*,*V*), and the posterodorsal subnucleus of the medial amygdala (MePD; Fig. 2*C*,*D*,*J*,*K*) as well as the premammillary nucleus of the hypothalamus (PMH) (Fig. 2*E*,*L*,*S*,*X*) and the retromammillary nucleus (RMN) (Fig. 2*E*,*L*) all contained tdTOM-positive cell bodies in both tdTom^DAT-Cre^ and tdTom^DAT-CreERT2^ mice. Within the lateral septum and RMN in particular, tdTOM was substantially more abundant in tdTom^DAT-CreERT2^ mice than tdTom^DAT-Cre^ mice (Fig. 2*A*,*E*,*H*,*L*). tdTOM was also identified in the dorsal raphe (Fig. 2*G*,*N*). These findings were further explored for validation.

### Clusters of mCHERRY-positive cell bodies confirm findings observed with tdTOM

**Figure 3.**
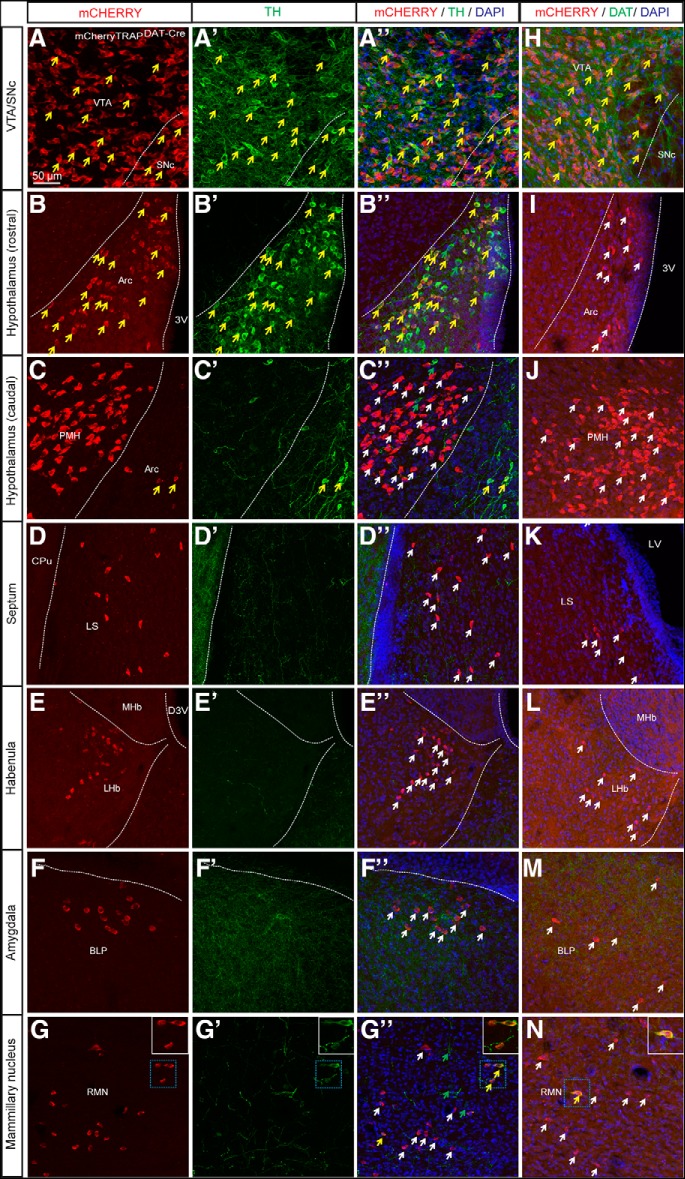
Clusters of mCHERRY-positive cell bodies confirm findings observed with tdTOM. Expression of mCHERRY-positive cell bodies in combination with (***A–G***) TH and (***H–N***) DAT immunofluorescence in the (***A***, ***H***) midbrain: VTA and SNc (***B***, ***I***) the arcuate nucleus and (***C***, ***J***) the PMH (***D***, ***K***) the lateral septum (***E***, ***L***) the LHb (***F***, ***M***) the BLP and (***G***, ***N***) the RMN of mCherryTRAP^DAT-Cre^ mice (white arrows showing mCHERRY-positive cell bodies, green arrows indicating TH-positive cells, and yellow arrows illustrating examples of co-localization; scale bar: 50 μm). See also Extended Data Figure 3-1. Arc, arcuate nucleus; CPu, caudate putamen; D3V, dorsal third ventricle; LS, lateral septum; LV, lateral ventricle; MHb, medial habenula; 3V, third ventricle Arc, Arcuate nucleus; CPu, Caudate putamen; D3V, Dorsal 3^rd^ ventricle; LHb, Lateral habenula; LS, Lateral septum; LV, Lateral ventricle; BLP, Posterior part of basolateral amygdala; MHb, Medial habenula; PMH, Premammillary nucleus; RMN; Retromammillary; SNc, Substantia nigra pars compacta; VTA: Ventral tegmental area; 3V, Third ventricle.

To validate that the identification of tdTOM protein in neurons classically not regarded as dopaminergic was specifically dependent of Cre activity, and not of non-Cre-dependent expression of the tdTom reporter line, the DAT-Cre mouse line was crossed with a second reporter line, mCherryTRAP. Analysis of direct fluorescence of the mCHERRY reporter in mCherryTRAP^*DAT-Cre*^ mice showed similar results as obtained with the tdTOM reporter above. mCHERRY-positive cell bodies were detected in the VTA and SNc and in the arcuate nucleus of the hypothalamus, validating the recombinatorial activity in dopaminergic areas. However, similar as described above, mCHERRY-positive cells were also seen within the lateral septum, the PMH, the LHb, distinct amygdalold subnuclei (BLP and MePD) and the RMN (Fig. 3*A–G*). Combination of mCHERRY reporter fluorescence with TH (Fig. 3*A’–G’*, *A’’–G’’*) and DAT immunoreactivity (Fig. 3*H–N*; Extended Data Fig. 3-1) showed the expected co-localization of mCHERRY and TH in midbrain (Fig. 3*A’’*) and the arcuate nucleus (Fig. 3*D’’*) as well as co-localization of mCHERRY and DAT in the midbrain (Fig. 3*H*). TH- and DAT-positive neuronal projections were seen innervating the BLP, but, as expected, no cell soma was immunopositive for TH or DAT in this cell cluster (Fig. 3*F’’*,*M*). None of the mCHERRY-positive cells of the PMH (Fig. 3*C*), the lateral septum (Fig. 3*D*) or the LHb (Fig. 3*E*) were immunopositive for either TH (Fig. 3*C’’–E’’*) or DAT (Fig. 3*J–L*) although a low degree of co-localization between mCHERRY and TH or DAT proteins was seen in the RMN (Fig. 3*G’’*,*N*).

10.1523/ENEURO.0198-19.2019.f3-1 Extended Data Figure 3-1 Rostrocaudal immunohistological analysis of mCHERRY and DAT proteins mCherryTRAP DAT-Cre mice. Immunofluoresence analysis showing detection of mCHERRY (red) and DAT (green) proteins (scale bar: 5 mm) Download Figure 3-1, TIF file.

### A subset of the DAT-Cre neurons of the PMH, LHb, and RMN are immunopositive for CALB1 and CALB2 proteins

**Figure 4.**
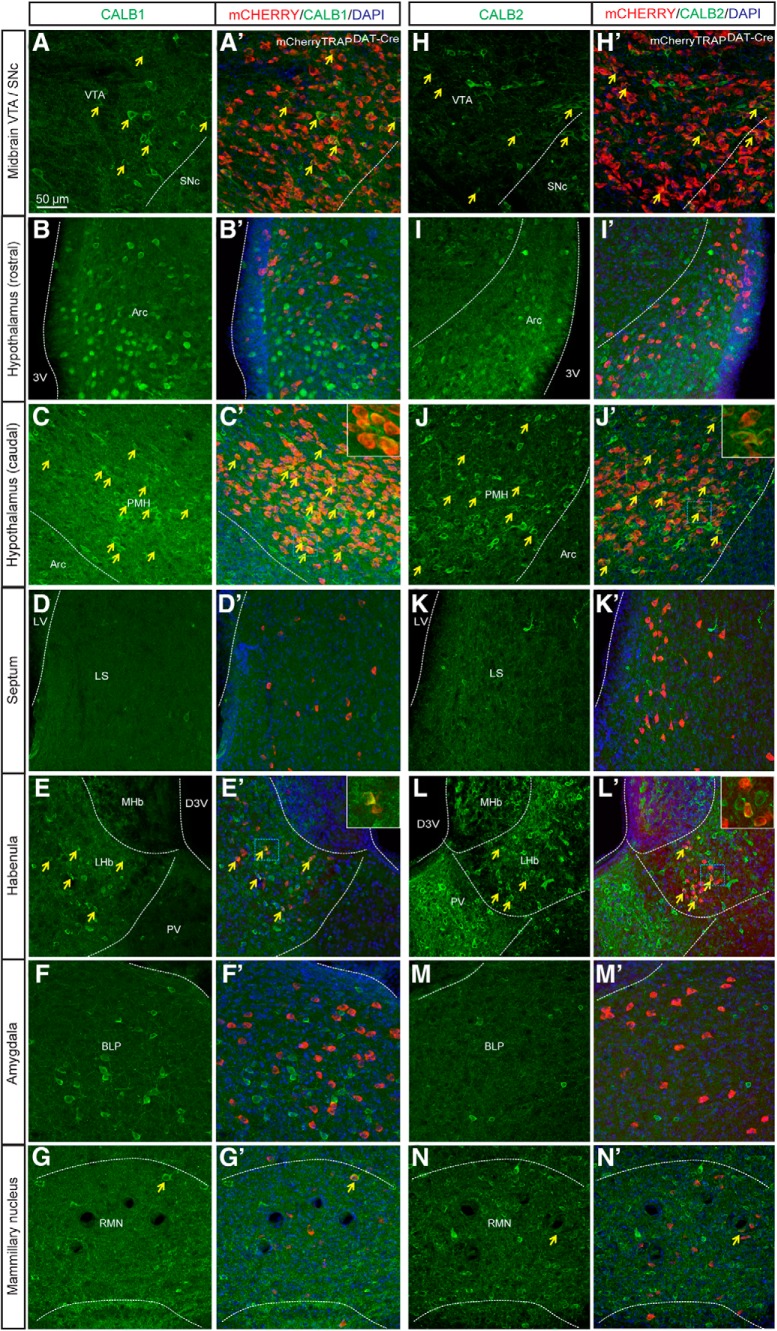
A subset of mCHERRY-positive cell bodies in the midbrain, the LHb, the PMH, and the RMN are immuno-positive for CALBINDIN (CALB1) and CALRETININ (CALB2). Immunofluorescent histological analysis of (***A–G***) mCHERRY (red) and CALB1 (green) and (***H–N***) mCHERRY (red) and CALB2 (green) in the (***A***, ***H***) midbrain, (***B***, ***I***) arcuate nucleus, and (***C***, ***J***) premammillary of the hypothalamus (PMH) (***D***, ***K***), the lateral septum, (***E***, ***L***) LHb, (***F***, ***M***) BLP of the amygdala, and (***G***, ***N***) RMN (yellow arrows illustrate example of cells where co-localization is detected; scale bar: 50 μm). Arc, arcuate nucleus; D3V, dorsal third ventricle; LS, lateral septum; MHb, medial habenula; PV, paraventricular thalamic nucleus; 3V, third ventricle; Arc, Arcuate nucleus; BLP, Basolateral amygdaloid nucleus posterior division; D3V, Dorsal third ventricle; LHb, Lateral habenula; LS, Lateral septum; MHb, Medial habenula; PMH, Premammillary nucleus of the hypothalamus; PV, Paraventricular thalamic nucleus; RMN, Retromammillary; SNc, Substantia nigra pars compacta; VTA: Ventral tegmental area; 3V, Third ventricle.

To characterize the molecular identity of the DAT-Cre neurons, we next performed a double immunohistological analysis using antibodies to detect the calcium-binding proteins CALBINDIN (CALB1; Fig. 4*A–G*) and CALRETININ (CALB2; Fig. 4*H–N*) in brain sections from mCherryTRAP^DAT-Cre^ mice. Co-localization between mCHERRY and CALB1 and CALB2 was observed in the VTA and SNc (Fig. 4*A’*,*H’*). In the hypothalamus, some mCHERRY-positive cells in the PMH, but not in the arcuate nucleus, were immuno-positive for CALB1 (Fig. 4*B’*,*C’*) and CALB2 (Fig. 4*I’*,*J’*). Similarly, while no co-localization between mCHERRY and CALB1 or CALB2 was detected in the lateral septum or in the amygdala (Fig. 4*D’*,*F’*,*K’*,*M’*), a proportion of mCHERRY-positive neurons in the LHb and RMN showed co-localization with CALB1 (Fig. 4*E’*,*G’*) and CALB2 (Fig. 4*L’*,*N’*).

### DAT-Cre-driven tdTom mRNA co-localizes extensively with Vglut2 or Gad1 mRNA

**Figure 5.**
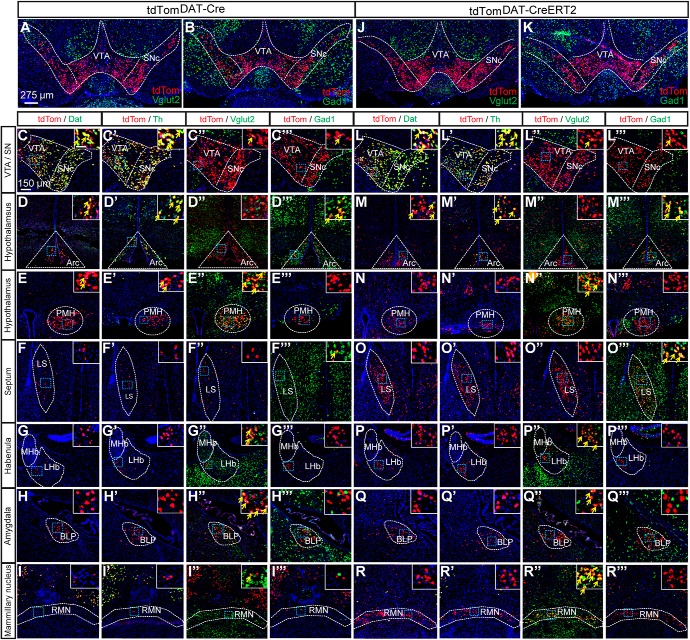
Double-FISH analyses of tdTom mRNA with Dat, Th, Vglut2, and Gad1 mRNAs identify multiple sites of tdTom/Vglut2 and tdTom/Gad1 double-positive cells in the brain of tdTom^DAT-Cre^ and tdTom^DAT-CreERT2^ mice. Double-FISH analysis of (***A***) tdTom and Vglut2 mRNAs or (***B***) tdTom and Gad1 mRNAs in the midbrain of tdTom^DAT-Cre^ mice and (***J***, ***K***) tdTom^DAT-CreERT2^ mice. Higher magnification of tdTom/Dat, tdTom/Th, tdTom/Vglut2, and tdTom/Gad1 mRNA within (***C–C’’’***) the VTA and SN (***D–D’’’***) the arcuate nucleus (**E*–E’’’***) and the PMH, (***F’–F’’’***) in the lateral septum (***G–G’’’***) the LHb, **(*H–H’’’***) the basal amygdala, (***I–I’’’***) and the RMN of tdTom^DAT-Cre^ mice. ***L–T’’’***, Similar expression analysis was performed in the tdTom^DAT-CreERT2^ mice for (***L–T***) tdTom/Dat, (***L’–T’***) tdTom/Th, (***L’’–T’’***) tdTom/Vglut2, and (***L’’’–T’’’***) tdTom/Gad1 mRNA in the different regions (yellow arrows indicate examples of colocalization between tdTom and Dat, Th, Vglut2, or Gad1 mRNA; scale bars: 275 and 150 μm; insets: 50 μm). Arc, arcuate nucleus; LS, lateral septum, MHb, medial habenula; SN, substantia nigra; Arcuate nucleus; BLP, Basolateral amygdaloid nucleus posterior division; LHb, Lateral habenula; LS, Lateral septum, MHb, Medial habenula; PMH, Premammillary nucleus of the hypothalamus, RMN, Retromammillary nucleus; SN, Substantia nigra, SNc, Substantia nigra pars compacta; VTA, Ventral tegmental area.

To systematically address the neurotransmitter identity of the observed *DAT-Cre* neurons, we next performed an extensive sdFISH experiment on serial sections derived from the entire brains of tdTom^*DAT-Cre*^ (Fig. 5*A–I’’’*; data summarized in Table 2) and tdTom^*DAT-CreERT2*^ mice (Fig. 5*J–R’’’*). In co-localization experiments, tdTom mRNA was compared to Dat, Th, Vglut2 and Gad1 mRNAs, of which the latter two served for the detection of glutamatergic and GABAergic identity, respectively.

**Table 2.** Summary of results obtained in double-FISH analysis of tdTom^DAT-Cre^ mice

Brain area		tdTom	Dat	Th	Vglut2	Gad1
SNc	Substantia nigra	+++	100%	100%	-	-
VTA	Ventral tegmental area	+++	60%	100%	7%	0.5%
RLi	Rostral linear raphe nucleus	++	20%	100%	70%	1%
CLi	Caudal linear caudal raphe	++	25%	100%	3%	-
GI	Glomerular layer of the olfactory bulb	+++	-	100%	-	100%
AO	Anterior olfactory area	+	-	-	-	-
LS	Lateral septum	+	-	-	-	100%
LHb	Lateral habenula	+	-	-	100%	-
Pe	Periventricular hypothalamic nucleus	+	-	-	-	8%
MPA	Medial preoptic area	0/+	-	-	-	-
CeA	Central amygdala	0/+	-	-	-	60%
BNST	Bed nucleus stria terminalis	+	-	-	-	100%
MePD/PV	Medial amygdala posterior part	+	-	-	-	100%
LH	Lateral hypothalamus	+	-	55%	-	100%
ADP	Anterodorsal preoptic nucleus	+	-	50%	-	100%
Arc	Arcuate nucleus of the hypothalamus	+++	100%	100%	-	100%
BLP	Basolateral amygdala posterior part	++	-	-	45%	1%
PMCo/AHiAL	Cortico/hip amygdala	+	-	-	100%	-
PMH	Premammillary nucleus of the hypothalamus	+++	5% weak signal	10% weak signal	100%	-
RMN	Retromammillary nucleus	+	-	-	100%	-
prEW/EW	Edinger–Westphal nucleus	+	-	100%	10%	-
PAG	Periaqueductal gray	++	-	100%	40%	-
RRF	Retrorubral field	+++	100%	100%	-	-
DR	Dorsal raphe	+	1%	100%	-	-
IC	Inferior colliculus	+	-	-	-	-
Cb	Purkinje cells of cerebellum	+++	-	-	-	100%
MPB	Medial parabrachial nucleus	+/++	-	5%	30%	100%
RtTg	Reticulotegmental nucleus of the pons	+/++	-	-	-	-

Semi-quantitative analysis of the degree of co-localization between tdTom mRNA (graded as +, ++, +++ for increasing density of positive cells) and Dat, Th, Vglut2, and Gad1 mRNAs, respectively. Representative images shown in Figure 5.

#### tdTom mRNA localization in brain of *tdTom^DAT-Cre^* mice

In tdTom^DAT-Cre^ mice, tdTom mRNA-positive neurons were as expected found in the VTA, SNc, and RRF of the ventral midbrain and in the arcuate nucleus of the hypothalamus (Fig. 5*A–D*). In addition, ample tdTom mRNA-positive neurons were identified in: The PMH (Fig. 5*E*) and BLP (Fig. 5*H*), and also in the PAG, glomerular layer of the olfactory bulb, the choroid plexus, and the Purkinje cells of the cerebellum (Table 2). At lower density, tdTom mRNA-positive neurons were found in the lateral septum (Fig. 5*F*), LHb (Fig. 5*G*), and RMN (Fig. 5*I*) and also in the MePD of the amygdala, the anterior olfactory area, the periventricular hypothalamic nucleus, medial preoptic area, central amygdala (CeA), bed nucleus of stria terminalis (BNST), lateral hypothalamus, anterodorsal preoptic area, posteromedial cortical amygdala, the anterolateral part of the amygdalohippocampal area (PMCo/AhiAL), Edinger–Westphal nucleus, dorsal raphe, inferior colliculus, medial parabrachial nucleus, and the reticulotegmental pontine nucleus (Table 2).

### Quantitative histological analysis of tdTom mRNA co-localization with Dat, Th, Vglut2, and Gad1 mRNAs in brain of tdTom^DAT-Cre^ mice

#### tdTom/Dat mRNA

Histological analysis of tdTom and Dat mRNA co-localization next showed that tdTom mRNA overlapped extensively with Dat mRNA in the SNc, VTA, RRF, RLi, and CLi as well as in the arcuate nucleus of the hypothalamus (Fig. 5*A–D*; Table 2). Quantification showed this tdTom/Dat mRNA overlap to be 100% in the SNc, RRF and arcuate nucleus, followed by lower degree of co-localization in the VTA (60%), RLi (20%), CLi (25%). Few cells in the PMH (5%) and dorsal raphe (1%) also showed co-localization (Table 2). None of the multiple additional areas in which tdTom was identified was positive for Dat mRNA (Table 2).

#### tdTom/Th mRNA

Quantitative histological analysis of co-localization between tdTom and Th mRNAs identified their overlap in several areas. tdTom mRNA co-localized to 100% with Th in the following DA-producing areas: VTA, SNc, RRF, RLi, CLi, arcuate hypothalamic nucleus (Fig. 5*A’–D’*; Table 2), glomerular cell layer of the olfactory bulb, PAG, and dorsal raphe (Table 2); 100% co-localization of tdTom and Th was also observed in the Edinger–Westphal nucleus (Table 2). In the lateral hypothalamus and anterodorsal preoptic nucleus, 55% and 50% of TdTom-positive neurons were also positive for Th mRNA while an observed tdTom/Th overlap was lower in the PMH (10%, weak signal only) and the medial parabrachial nucleus (5%; Table 2).

#### tdTom/Vglut2 mRNA

Also, quantitative histological analysis of co-localization between tdTom and Vglut2 mRNAs identified overlap in several areas. tdTom mRNA co-localized to 100% with Vglut2 mRNA in the PMH (Fig. 5*E’’*; Table 2), LHb (Fig. 5*G’’*; Table 2), RMN (Fig. 5*I’’*; Table 2) and in the anterolateral part of the amygdalohippocampal area (PMCo/AhiAL; Table 2). tdTom/Vglut2 co-localization was also observed in the following areas: VTA (7%), RLi (70%), CLi (3%), BLP (45%), Edinger–Westphal nucleus (10%), PAG (40%), and medial parabrachial nucleus (30%).

#### tdTom/Gad1 mRNA

Several regions showed substantial overlap between tdTom and Gad1 mRNAs; 100% tdTom/Gad1 co-localization was observed in the following areas: lateral septum (Fig. 5*F’’’*; Table 2), arcuate nucleus (Fig. 5*D’’’*; Table 2), glomerular layer of the olfactory bulb, BNST, MePD, lateral hypothalamus, anterodorsal preoptic nucleus, Purkinje cells of the cerebellum, and the medial prebrachial nucleus (Table 2). Lower degree of overlap was observed in the VTA (0.5%), RLi (1%), periventricular hypothalamic nucleus (8%), CeA (60%), and the BLP (1%; Table 2).

#### Comparison between several *mRNAs*

Out of all areas that were 100% positive for co-localization of tdTom and Th, only the SNc, RRF, and arcuate nucleus were also 100% positive for Dat mRNA (Table 2). Further, none of the areas that were positive for Dat mRNA were negative for Th mRNA (Table 2). However, some of the Th-positive neurons were also positive for Vglut2 or Gad1 mRNA. In areas 100% positive for Th, tdTom/Vglut2 mRNA double positive neurons were found in VTA (including RLi, CLi), SNc, PAG, medial parabrachial nucleus, and the Edinger–Westphal nucleus (Table 2). Further, in the arcuate nucleus and the glomerular layer of the olfactory bulb, 100% of the tdTom cells were positive for both Th and Gad1 mRNAs. In the arcuate nucleus, all tdTom cells were also positive for Dat mRNA while negative for Vglut2, thus showing a tdTom/Th/Dat/Gad1 phenotype (Table 2).

### tdTom mRNA localization in brain of *tdTom^DAT-CreERT2^* mice

Similar findings as described above were obtained in the tdTom^DATCre-ERT2^ line (Fig. 5*J–R*) with high co-localization of tdTom and Vglut2 mRNAs in the LHb, PMH and RMN (Fig. 5*N’’*,*P’’*,*R’’’*) and high degree of co-localization of tdTom and Gad1 mRNAs in the lateral septum (Fig. 5*O’’’*). In the amygdala, tdTom mRNA co-localized abundantly with Vglut2 mRNA in the BLP (Fig. 5*Q’’*) and with Gad1 mRNA in the MePD (Table 2). The number of tdTom-positive cells was substantially higher in all regions of the amygdala in the tdTom^DATCre-ERT2^ line compared to the DAT-Cre line.

### Comparing current histological results with data published by the Allen Brain Institute enables validation of ectopic DAT-Cre-driven gene expression in multiple brain areas

Next, areas identified above in the histological analyses as positive for DAT protein (Fig. 3), Dat mRNA (Fig. 5), tdTOM protein (Fig. 3), and tdTom mRNA (Fig. 5; Table 2) in the current tdTom^DAT-Cre^ mouse line were summarized (Table 3). As evident from above, among all tdTom-positive areas, only areas established to produce DA were positive for Dat mRNA while all other tdTom-positive neurons were negative Dat mRNA (Fig. 5; Table 3). Data of tdTom mRNA were subsequently compared with analyses reported in the Allen Brain Atlas in which another DAT-Cre mouse line, the “*Slc6a3-Cre*” line (Zhuang et al., 2005) has been characterized using three different *tdTom* lines (Ai9, Ai14, and Ai34; Madisen et al., 2010; Abraira et al., 2017) and the Cre-dependent TET controllable egfp reporter *Ai148^(TIT2L-GC6f-ICL-tTA2)^* (Daigle et al., 2018). Upon comparison with data obtained with Ai14 and Ai9 reporter lines (experiments #100138615 and #81439487), an almost complete match between the current findings and those reported in Allen Brain Atlas was found (summarized in Table 3). There was a slight discrepancy in detection of tdTom mRNA in the striatal complex and median eminence which was only very rarely detected by mRNA in the present study, but which was reported for the “Slca3-Cre” line (Table 3). Apart from this, all areas detected as positive for tdTom were the same between the two Cre-lines, thus confirming activity of Cre recombinase in both dopaminergic and non-dopaminergic brain areas.

**Table 3.**
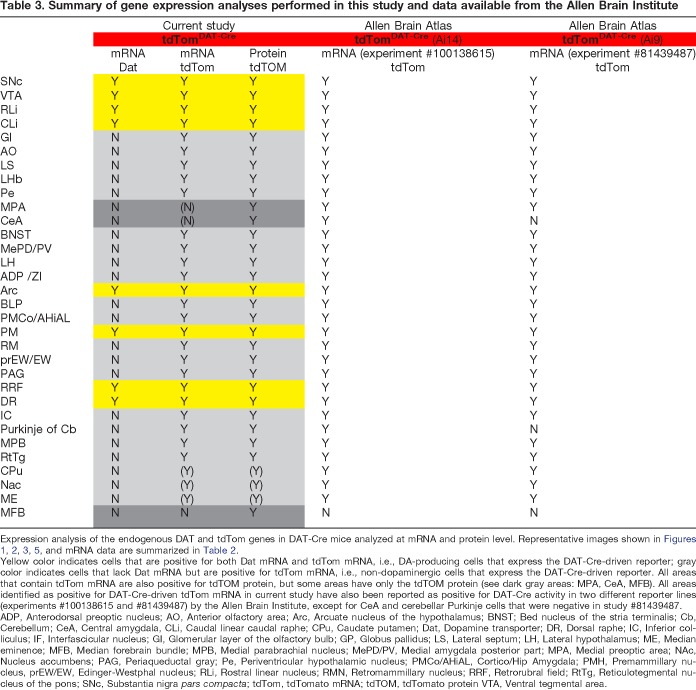
Summary of gene expression analyses performed in this study and data available from the Allen Brain Institute

Expression analysis of the endogenous DAT and tdTom genes in DAT-Cre mice analyzed at mRNA and protein level. Representative images shown in Figures 1, 2, 3, 5, and mRNA data are summarized in Table 2.

Yellow color indicates cells that are positive for both Dat mRNA and tdTom mRNA, i.e., DA-producing cells that express the DAT-Cre-driven reporter; gray color indicates cells that lack Dat mRNA but are positive for tdTom mRNA, i.e., non-dopaminergic cells that express the DAT-Cre-driven reporter. All areas that contain tdTom mRNA are also positive for tdTOM protein, but some areas have only the tdTOM protein (see dark gray areas: MPA, CeA, MFB). All areas identified as positive for DAT-Cre-driven tdTom mRNA in current study have also been reported as positive for DAT-Cre activity in two different reporter lines (experiments #100138615 and #81439487) by the Allen Brain Institute, except for CeA and cerebellar Purkinje cells that were negative in study #81439487.

ADP, Anterodorsal preoptic nucleus; AO, Anterior olfactory area; Arc, Arcuate nucleus of the hypothalamus; BNST; Bed nucleus of the stria terminalis; Cb, Cerebellum; CeA, Central amygdala, CLi, Caudal linear caudal raphe; CPu, Caudate putamen; Dat, Dopamine transporter; DR, Dorsal raphe; IC, Inferior colliculus; IF, Interfascicular nucleus; GI, Glomerular layer of the olfactory bulb; GP, Globus pallidus; LS, Lateral septum; LH, Lateral hypothalamus; ME, Median eminence; MFB, Median forebrain bundle; MPB, Medial parabrachial nucleus; MePD/PV, Medial amygdala posterior part; MPA, Medial preoptic area; NAc, Nucleus accumbens; PAG, Periaqueductal gray; Pe, Periventricular hypothalamic nucleus; PMCo/AHiAL, Cortico/Hip Amygdala; PMH, Premammillary nucleus, prEW/EW, Edinger-Westphal nucleus; RLi, Rostral linear nucleus; RMN, Retromammillary nucleus; RRF, Retrorubral field; RtTg, Reticulotegmental nucleus of the pons; SNc, Substantia nigra *pars compacta*; tdTom, tdTomato mRNA; tdTOM, tdTomato protein VTA, Ventral tegmental area.

### The DAT-Cre transgene is active in neurons of the LHb and amygdala of the adult mouse which enables identification of neuron-specific target areas

**Figure 6.**
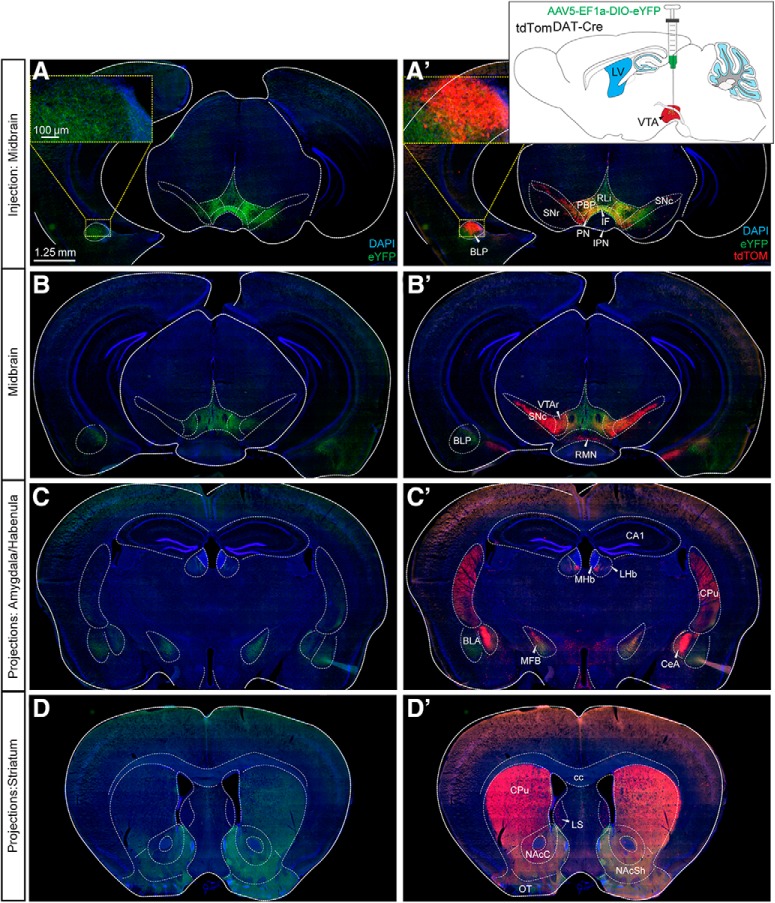
Intracranial injection of AAV5-eYFP into the VTA of tdTom^DAT-Cre^ mice verifies activity of Cre recombinase and enables identification of projections. ***A***, ***A’***, ***B***, ***B’***, Stereotaxic viral injections of AAV5-EF1a-DIO-eYFP into the VTA of tdTom^DAT-Cre^ mice. Schematic illustration shows the injection site (***A’***). eYFP (***A–D***); eYFP and tdTOM (***A’–D’***). ***A***, ***A’*** and ***B***, ***B’***, Two different rostro-caudal levels of the midbrain. Cytoplasmic eYFP (green) detected in the VTA and some also in the SNc (***A***, ***B***) with eYFP-positive projections detected in the BLP (in the section level shown in ***A***). tdTOM-positive cells in VTA and SNc (***A’***, ***B’***); also, the tdTOM-positive cell cluster shown in Figure 1 is detected in the BLP at the section level of ***A*** (***A’***; inset showing tdTOM-positive cell cluster and eYFP-positive fibers in the BLP, ***A’***). ***C***, ***C’***, ***D***, ***D’***, eYFP-positive projections in additional target structures known for the VTA and SNc: the BLA and CeA of the amygdala and the habenula (***C–C’***), and the dorsal and ventral aspects of the striatum (***D–D’***). At this section level, TdTOM is detected in the CeA (scale bar: 1.25 mm; inset: 100 μm). See also Extended Data Figure 6-1. BLA, basolateral amygdala; CA1, CA1 region of hippocampus; cc, corpus callosum; CPu, caudate putamen; IPN, interpeducular nucleus; LS, lateral septum; LV, lateral ventricle; MFB, medial forebrain bundle; MHb, medial habenula; NAcSh, NAc shell; OT, olfactory tubercle; VTAr, VTA rostral part.

**Figure 7.**
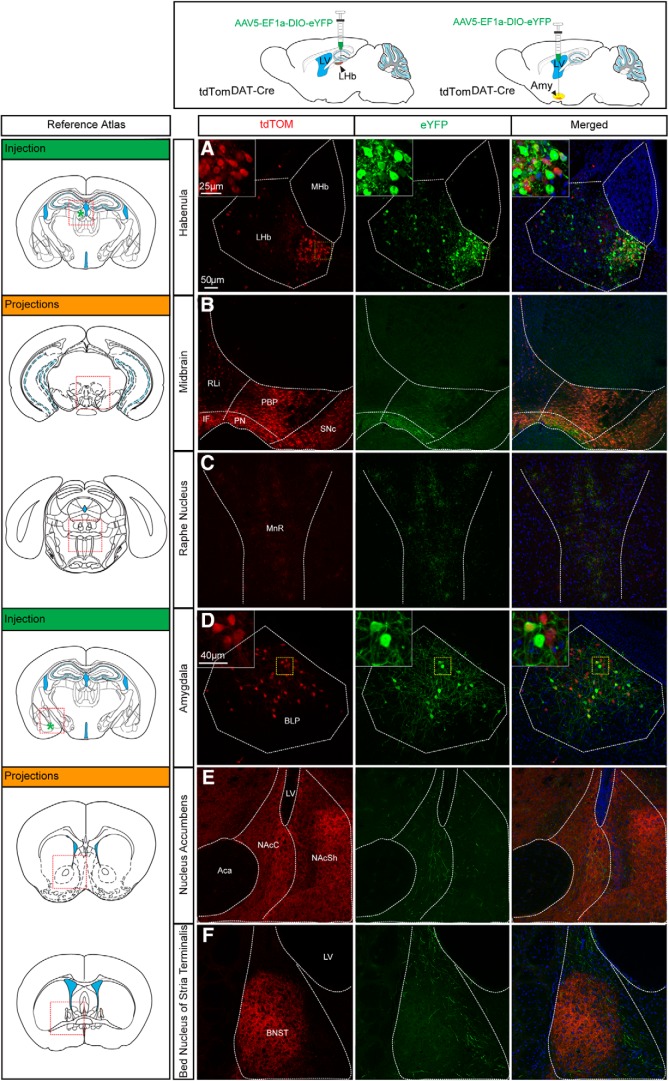
Intracranial injections of AAV5-eYFP into the LHb and into the amygdala of tdTom^DAT-Cre^ mice verify Cre activity in these areas, and enable identification of distinct projections from each of these areas. ***A***, Stereotaxic injections with AAV5-EF1a-DIO-eYFP into the LHb of tdTom^DAT-Cre^ mice. eYFP (green) and tdTOM (red) immunofluorescent-positive cell bodies of the LHb; eYFP (green)-positive projections detected in (***B***) the medioventral VTA, and (***C***) in the median raphe nucleus. ***D***, Stereotaxic injections with AAV5-EF1a-DIO-eYFP into the LHb of tdTom^DAT-Cre^ mice. eYFP (green) and tdTOM (red) immunofluorescent-positive cell bodies in the BLP of amygdala and eYFP-positive projections in (***E***) the ventral striatum (NAcC) and (***F***) the BNST (scale bar: 50 μm; inset: 25 and 40 μm). Aca, anterior commissure, anterior part; LV, lateral ventricle; MHb, medial habenula; MnR, medial raphe nucleus, NAcSh, NAc shell; SN, substantia nigra.

**Figure 8.**
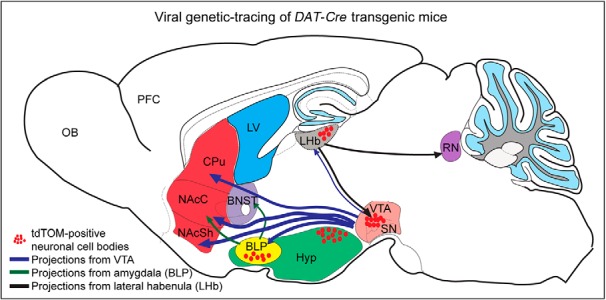
Schematic drawing illustrating projections from amygdala and LHb seen by viral-genetic tracing of non-dopaminergic DAT-Cre neurons identified in the study. VTA injections used as control. CPu, caudate putamen; LV, lateral ventricle; NAcSh, NAc shell; OB, olfactory bulb; PFC, prefrontal cortex; RN, raphe nucleus; SN, substantia nigra Hyp, Hypothalamus; NAcC, NAc core.

Having identified DAT-Cre-dependent reporter gene expression in both dopaminergic and non-dopaminergic brain areas, a viral-genetic approach was next implemented to further verify that the DAT-Cre transgene is active in the adult mouse. For this purpose, an *AAV5-EF1a-DIO-eYFP* virus was stereotactically injected into the VTA, LHb, and amygdala of tdTom^DAT-Cre^ mice. DAT-Cre-dependent expression of the floxed eYFP reporter in the VTA has in several publications solidly been shown to give rise to eYFP labeling in the VTA, and to co-localize with TH and DAT immunoreactivity (Lammel et al., 2015; Pascoli et al., 2015). VTA-injections were therefore used as controls. As expected, injection of the virus into the VTA gave rise to robust expression of eYFP in the VTA and to some expression also in the laterally located SNc (Fig. 6*A–B’*). The floxed reporter (eYFP) showed complete overlap with the inherited reporter (tdTOM), however, due to the spatial restriction of the viral injection, not all tdTOM-positive cells had eYFP. Strong eYFP-positive projections and fibers were observed in established target areas of VTA DA neurons, primarily the ventral striatum, also known as the nucleus accumbens (NAc) shell and also in the NAc core (NAcC) and the basolateral subarea of the amygdaloid complex, including the BLP (Fig. 6*A–D*). Projections within the BLP were detected within a cluster that also contained tdTOM-positive cell bodies (Fig. 6*A*). The dorsal aspect of the striatum also contained some eYFP-positive fibers, however, at a much lower density than the ventral striatum (Fig. 6*D*). In addition, eYFP-positive fibers were detected along the median forebrain bundle and in the olfactory tubercle, intermediate part of the lateral septum, BNST, ventral pallidum, LHb, RRF, raphe nucleus and CLi of the caudal VTA (Extended Data Fig. 6-1). Upon injection of the *AAV5-EF1a-DIO-eYFP* virus into the LHb of *tdTom^DAT-Cre^*, robust YFP fluorescence was observed within tdTOM-positive neurons in this area. Immunoreactivity for the eYFP and tdTOM reporters showed substantial overlap (Fig. 7*A*). When addressing the distribution of eYFP-positive projections on the injection into LHb, these were detected in the PN and IF of the VTA and in the raphe nucleus, in particular the medial raphe (Fig. 7*B*,*C*). Upon injection of *AAV5-EF1a-DIO-eYFP* into the amygdala, eYFP and tdTOM double-positive immunoreactivity was observed with restricted eYFP-positive projections to the NAcC and the BNST (Fig. 7*D*). These viral-genetic experiments enabled identification of projections from Cre-expressing dopaminergic (VTA, control) and non-dopaminergic (LHb and amygdala) neurons and thus also verified that Cre recombinase is active in these areas in adult mice. A summary of the identified projections from DAT-Cre-positive neurons in the LHb and amygdala is shown in Figure 8. Table 4 lists all abbreviations used for anatomical areas throughout the text and in figures.

10.1523/ENEURO.0198-19.2019.f6-1 Extended Data Figure 6-1 Intracranial injections of AAV5-eYFP into the VTA of tdTomDAT-Cre mice. ***A–C***, Immunofluorescence analysis of eYFP (green) and tdTOM (red) proteins along the rostrocaudal axis (scale bar: 2.5 mm). Download Figure 6-1, TIF file.

**Table 4.** Abbreviations of anatomical regions used in text and/or figures

**Anatomical region**	**Abbreviation**	**Anatomical region**	**Abbreviation**
Anterior commissure	Aca	Medial forebrain bundle	MFB
Anterodorsal preoptic nucleus	ADP	Medial habenula	mHb
Anterior olfactory area	AO	Medial preoptic area	MPA
Arcuate nucleus of the hypothalamus	Arc	Medial parabrachial nucleus	MPB
Basolateral amygdala	BLA	Medial raphe nucleus	MnR
Basolateral amygdala posterior part	BLP	Nucleus accumbens core	NacC
Bed nucleus stria terminalis	BNST	Nucleus accumbens shell	NAcSH
CA1 region of hippocampus	CA1	Olfactory tubercle	OT
CA3 region of hippocampus	CA3	Periaqueductal gray	PAG
Cerebellum	Cb	Parabrachial pigmented area	PBP
Corpus callosum	cc	Periventricular hypothalamic nucleus	Pe
Central amygdala	CeA	Prefrontal cortex	PFC
Caudal linear raphe nucleus	CLi	Cortico/Hip amygdala	PMCo/AHiAL
Caudate putamen	Cpu	Premammillary nucleus of the hypothalamus	PMH
Dopamine	DA	Retromammillary nucleus	RMN
Dorsal raphe	DR	Paranigral nucleus	PN
Dorsal aspect third ventricle	D3V	Edinger-Westphal nucleus	prEW/EW
Glomerular layer of the olfactory bulb	GI	Rostral linear raphe nucleus	RLi
Hypothalamus	Hyp	Raphe nucleus	RN
Inferior colliculus	IC	Retrorubral field	RRF
Interfascicular nucleus	IF	Reticulotegmental nucleus of the pons	RtTg
Interpeduncular nucleus	IPN	Substantia nigra pars compacta	SNc
Lateral habenula	LHb	Substantia nigra pars reticulata	SNr
Lateral hypothalamus	LH	Subthalamic nucleus	STN
Lateral septum	LS	Ventral tegmental area	VTA
Lateral ventricle	LV	Zona incerta	ZI
Median emminence	ME	Third ventricle	3V
Medial amygdala posterior part	MePD/PV		

## Discussion

In this study, we report the observation of DAT-Cre-driven reporter gene expression within cell clusters of the septum, habenula and amygdala as well as multiple additional brain sites, none of which is commonly associated with expression of the endogenous *Dat* gene. In both DAT-Cre and DAT-CreERT2 mice, this seemingly ectopic reporter gene expression was made using the *Ai14* reporter line in which the reporter, the tdTOM fluorescent protein, labels both cell bodies and projections. Similar findings were subsequently obtained when crossing the DAT-Cre transgene with the *mCherryTRAP* reporter line in which fluorescent mCHERRY labels cell bodies only. In addition to coherent results obtained from these two different reporters and absence of reporter gene expression in the absence of Cre recombinase, the findings were robustly confirmed by comparison with data published in the Allen Brain Atlas. Further, when addressing neurotransmitter identity of the ectopic *DAT-Cre* neurons, we identified that several of these were positive for Vglut2 or Gad1 mRNA, suggesting that *Dat* regulatory sequences, at least in transgenic constructs, can be turned on in glutamatergic and GABAergic neurons. For example, we identified that tdTom mRNA in the LHb, the BLP, the PMH, and the RMN colocalized with Vglut2 mRNA, while in the MePD and the lateral septum, tdTom mRNA showed substantial overlap with Gad1 mRNA. A modest subset of these nuclei was immunopositive for CALB1 and CALB2, further defining their molecular identity. Finally, we report that DAT-Cre-driven recombination in the amygdala and habenula gave rise to reporter gene expression on viral-genetic delivery of fluorescent reporter construct in adult mice, firmly demonstrating the activity of Cre recombinase in these non-dopaminergic neurons at the mature stage.

### Comparable reporter gene expression in several different DAT-Cre/Lox systems validates results

The *DAT-Cre* and *DAT-CreERT2* mice used in the present study were originally validated for Cre activity in midbrain DA neurons (Ekstrand et al., 2007; Engblom et al., 2008) and also implemented recently for the study of hypothalamic DA neurons (Soden et al., 2016; Stagkourakis et al., 2018b, 2019). Produced by different molecular and transgenic strategies, the DAT-Cre knock-in line by homologous recombination in the endogenous *Dat* locus (i.e., in this transgenic line, Cre is present in the endogenous *Dat* locus), and the DAT-CreERT2 transgenic line mice by random integration upon cloning of the Cre gene downstream of DAT promoter sequences available in a BAC-clone, both these DAT-Cre-drivers have been shown to faithfully give rise to Cre activity in *Dat*-expressing midbrain DA neurons (Ekstrand et al., 2007; Engblom et al., 2008). The findings obtained in the present study further validate that *mCherry* and *tdTom* reporter gene expression co-localizes with *Th* and *Dat* gene expression on both mRNA and protein level. Co-localization in the ventral midbrain was restricted to the dopaminergic populations of the VTA, SNc, and RRF and was not detected in neighboring structures of the ventral midbrain. This observation is in accordance with findings by Lammel and colleagues reporting greater selectivity for DA neurons in the DAT-Cre than the TH-Cre transgenic line (Lammel et al., 2015). Several studies using TH-Cre-knock-in mice have reported ectopic expression of Cre in midbrain precursor cells that never produce TH or lose their ability to do so on differentiation (Lindeberg et al., 2004; Savitt et al., 2005; Nordenankar et al., 2015a).

Our initial identification of seemingly ectopic reporter gene expression in the BLP in tdTom^DAT-Cre^ mice motivated us to systematically analyze whether this unexpected finding applied to additional brain areas. Since transgenic mice, including both Cre-driver lines and floxed reporters, may suffer from variability in expression of the transgene, we chose to address more than one DAT-Cre/Lox system to validate key histological findings. By analysis of different types of floxed reporters achieved by two different DAT-Cre transgenes and, by comparing the results with data available from the Allen Institute for Brain Science, the transgenic data presented here has been validated in several systems. Reporter lines have been shown to vary in their recombination efficiency (Madisen et al., 2010; Sun et al., 2014), and while the majority of findings were consistent between the different combinations of Cre/Lox systems addressed, also some differences were found. The spatial reporter gene expression in the DAT-Cre transgenice mouse line addressed in the current study identified in the lateral septum, PMH, LHb, and the BLP, CeA, and MePD of the amygdaloid complex is consistent with the characterization pattern available from the Allen Institute for the *Slc6a3(Dat)-Cre* mouse (Zhuang et al., 2005) as was also the reporter gene expression identified in multiple additional brain areas (summarized in Table 3). However, the RMN was only weakly detected when the *Slc6a3-Cre* line was crossed with the Ai9 (experiment #81439487) and Ai134 (experiment #111202482), while prominently expressed with the Ai148 line (experiment #571262267), in accordance with our findings using the tamoxifen-inducible *DAT-Cre-ERT2* line. In addition, while the different regions of the amygdaloid complex that expressed tdTom cells were similar between the different reporters, the higher number of cells detected in the *DAT-Cre-ERT2* line resembled that of the Allen Brain Institute using the Ai9 reporter. Importantly, with the exception of the arcuate nucleus and the PMH, in which Dat mRNA but not DAT protein was found, none of the other neuronal clusters outside the classical dopaminergic brain areas that were positive for tdTom mRNA or TdTOM protein in our study expressed the *Dat* gene. This is in slight contrast to the *in situ* data from the Allen Brain Institute that shows Dat mRNA in the LHb.

Comparing our results from tamoxifen-inducible *DAT-CreERT2* transgenic mice with the *DAT-Cre* knock-in mice, it was clear that stronger *tdTom* reporter expression was observed on recombination achieved by the tamoxifen-inducible DAT-CreERT2 than the DAT-Cre. Time of initiation of expression of the Cre recombinase (eight-week-old adult mice for DAT-CreERT2 mice versus embryonal development for DAT-Cre mice) might reflect the difference in intensity and amount of cells positive for the tdTom reporter. However, at large, the same areas were positive for the reporter no matter of age from which the Cre recombinase was present. This is particularly interesting considering that regulatory events during developmental stages might, at least in theory, induce both endogenous *Dat* gene expression and the DAT-Cre transgenic construct (which has been cloned into the endogenous Dat locus) in several areas leaving DAT-Cre to remain present despite a subsequent down-regulation of the endogenous *Dat* gene products in non-dopaminergic areas in the mature brain. This way, the presence of DAT-Cre in non-dopaminergic areas would not quite qualify as “ectopic,” as it would be regulated along with *Dat*, even if transient. However, considering that transcription from a transgenic DAT-Cre can be induced in the adult mouse, and evidently give rise to efficient recombination, a more likely explanation for the Cre-mediated recombination in areas devoid of endogenous *Dat* expression in the adult is that the promoter sequences used in any of the studied DAT-Cre-lines are sufficient to drive transgenic expression while leaving the endogenous *Dat* gene untranscribed or, at least, below detection level. While it remains to be fully solved how and why DAT-Cre transgenic constructs are expressed in areas not positive for endogenous *Dat* expression, the recombinatorial Cre activity of DAT-Cre transgenes should be useful as tool for the neurons that do show this unanticipated feature, for example in viral-genetic experiments.

### Neurotransmitter identity of ectopic DAT-Cre neurons

Based on double-labeling experiments using FISH, the current data show that DAT-Cre-positive neurons are found in a number of glutamatergic (e.g., LHb, BLP, PMH, RMN) and GABAergic (e.g., lateral septum, BNST, Purkinje layer of cerebellum) neurons, and also in neurons that are positive for more than one neurotransmitter marker. It is today well established that certain groups of neurons, such as within the VTA, have the capacity to co-release neurotransmitters (Trudeau et al., 2014; Pupe and Wallén-Mackenzie, 2015; Barker et al., 2016; Granger et al., 2017; Morales and Margolis, 2017). Subregions of the VTA have been reported to consist of neurons that express “TH only,” “Vglut2 only,” and “dual Vglut2/TH” markers (Morales and Root, 2014). In this study, we found tdTom positive cells that co-expressed Th and Vglut2 mRNAs in the Edinger–Westphal nucleus, the CLi and the PAG in addition to the VTA. Moreover, tdTom cells in the glomerular layer of the olfactory bulb co-expressed *Th* and *Gad1* genes, while the cells located in the arcuate nucleus of the hypothalamus were positive for Dat, Th and Gad1 mRNA. Further, while some of the tdTom-positive neuronal groups expressed one or more than one of the neurotransmitter markers used here (Dat, Th, Vglut2, Gad1), other areas were devoid of any of these mRNAs. While we selected Vglut2 mRNA as marker for glutamatergic neurons, VGLUT2 along with VGLUT1 and VGLUT3 are the vesicular transporters used by glutamatergic neurons to package glutamate (Fremeau et al., 2004). Here, we used Vglut2 mRNA to visualize how DAT-Cre neurons can be of glutamatergic identity, but our analysis does not claim to be exhaustive. VGLUT1 is abundant in forebrain neurons, and most glutamatergic neurons are positive for either Vglut1 or Vglut2 mRNA (and protein). This is particularly striking in amygdaloid subnuclei where Vglut1 and Vglut2 cover different subnuclei, and where Vglut1 is most prominent (Poulin et al., 2008; Wallén-Mackenzie et al., 2009; Nordenankar et al., 2015b). Similar to the need for analysis of different markers to fully describe a glutamatergic phenotype, also GABA neurons are represented by markers that differ in their expression. Here, we used a Gad1 probe for detection of GABA neurons, but for a complete analysis of DAT-Cre neurons using GABA as neurotransmitter, analysis of Gad2 mRNA could also have been included. Our semi-quantitative histological mapping of ectopic DAT-Cre neurons, despite using a limited set of neurotransmitter markers, identified glutamatergic, GABAergic and co-releasing neurons in the DAT-Cre population. In the event that any of these newly identified DAT-Cre cell clusters be used for neurocircuitry analysis, it would be necessary to perform a more detailed neurotransmitter analysis within the particular area of interest.

### Functional implications of ectopic DAT-Cre neurons for neurocircuitry analysis

Previous studies addressing DAT-Cre positive neurons in the arcuate nucleus and PMH have reported Dat or Th mRNA as well as TH and DAT immunoreactivity in these hypothalamic regions (Soden et al., 2016; Meng et al., 2018; Stagkourakis et al., 2018a). It has been shown that about half of the TH-positive neurons of the PMH of the hypothalamus express both Th and Vglut2 mRNA and undergo a neuronal switch on stress by decreasing in number, while the remaining TH-positive neurons are unaffected (Meng et al., 2018). In the present study, Vglut2 mRNA was seen in the majority of the PMH DAT-Cre neurons suggesting a glutamatergic nature of this population as reported previously in the rat hypothalamus (Ziegler et al., 2002). This is also in accordance with a recent study which reported glutamate release on optogenetic stimulation of the PMH DAT-Cre neurons in mice (Soden et al., 2016). Aside from the social and exploratory behavior of the PMH (Soden et al., 2016), the PMH together with the lateral septum and the amygdala form a circuity involved in innate behavior and aggression. Given that the DAT-Cre transgenic line has previously been used for investigating male aggression by targeting the ventral PMH DAT-Cre neurons by means of optogenetics (Stagkourakis et al., 2018a), it could be of interest to analyze whether the DAT-Cre-expressing cells located in the lateral septum and the amygdala play any role in this neuronal circuitry and innate behavior. Previous studies have shown that the GABAergic neurons in the medial amygdala, MePD, are involved in sexual experiences and social aggression (Hong et al., 2014; Li et al., 2017). As the DAT-Cre neurons in the amygdala were not positive for Th or Dat mRNA, but showed overlap between Gad1 and tdTom, it could be of interest to address whether the cluster of DAT-Cre cells in the MePD may be involved in similar behavioral manifestations by forming part of the neuronal network linking the lateral septum, amygdala, and hypothalamus.

In the present study, upon intracranial injection of AAV5-EF1a-DIO-eYFP in the DAT-Cre population of the basal amygdala, eYFP-positive projections were detected in the NAcC and the BNST. The NAcC and the BNST are two brain regions associated with anxiety and addiction (Di Chiara, 2002; Stamatakis et al., 2014; Avery et al., 2016; Vranjkovic et al., 2017; Yang et al., 2018a.) Previous studies have shown that the amygdala with its projections to the NAc and the BNST are involved in positive and negative valence. The pathway attributed to the behavioral outcome has been shown as glutamatergic, firstly by glutamate release directly regulating presynaptic DA release in the NAc, and secondly by the inputs from the amygdala indirectly controlling DA release by activating the projection neurons in the ventral striatum that form a feedback loop with the VTA (Stuber et al., 2011; Watabe-Uchida et al., 2012; Kim et al., 2013; Beyeler et al., 2016). Based on the co-expression of tdTom and Vglut2 mRNA in DAT-Cre neurons of the basal amygdala, and their projections to the BNST and the NAc, it would be of potential interest to explore the role in these DAT-Cre neurons in reinforcing behavior.

Along the same line, the LHb, in particular via the mesohabenular pathway, has attracted significant attention due to its role in addiction, reward, aversion, anxiety and depression (Matsumoto and Hikosaka, 2007; Hikosaka et al., 2008; Namboodiri et al., 2016; Fakhoury, 2017; Mizumori and Baker, 2017; Yang et al., 2018b). In this study, we were able to detect eYFP fibers in the LHb upon viral injection of *AAV5-EF1a-DIO-eYFP* in the VTA of the DAT-Cre mice, in accordance with previous findings (Stuber et al., 2015). Moreover, the LHb has been shown to influence aversive or reward processing and play a role in evaluating reward prediction errors. These have been linked to the LHb inputs on the dopaminergic neurons of the VTA and SN, the serotonergic neurons in the RN and the GABAergic neurons in the rostromedial tegmental nucleus (Lammel et al., 2012; Proulx et al., 2014; Quina et al., 2015; Zhao et al., 2015; Baker et al., 2016). Interestingly, the spatial location of the DAT-Cre neurons identified within the LHb resembles the regional distribution of the glutamatergic projections from the lateral hypothalamus to the LHb that project to the VTA and been reported to evoke aversion (Proulx et al., 2014; Mizumori and Baker, 2017; Lazaridis et al., 2019). This raises the possibility that the *DAT-Cre* expressing neurons in the LHb may have the same projections. Indeed, in addition to the finding that tdTom in the LHb colocalized with Vglut2 mRNA, viral delivery of a floxed eYFP reporter in the LHb also showed eYFP positive projections in the median raphe and in the VTA. Our investigation of non-dopaminergic DAT-Cre-positive neuronal clusters in the amygdala and LHb using viral-genetic methodology in adult mice had two goals: validate that Cre recombinase present in non-dopaminergic neurons could give rise to active recombination of floxed alleles in mature neurons, and address whether these Cre-expressing neurons might be of interest for further analysis and manipulation, here examplified by addressing if projection patterns from these neurons could be observed. Indeed, based on our results, we can demonstrate that Cre recombinase successfully targets floxed alleles in the LHb and amygdala of adult DAT-Cre mice, and also that this type of experiment allows for identification of projections and projection target areas for these DAT-Cre-positive, non-dopaminergic, neurons.

A limiting factor of the study might be the absence of viral-genetic approaches in wild-type mice to confirm that neurons of the amygdala and LHb do not give rise to recombination of floxed eYFP even in the absence of Cre recombinase. However, during our validations of optogenetics-based approaches using DAT-Cre mice in the context of viral-genetic approaches in the VTA (Bimpisidis et al., 2019), we have never observed any reporter gene expression in any brain area in mice negative for Cre recombinase.

In conclusion, in this study we identified robust DAT-Cre driven reporter gene expression in cell somata in multiple brain regions associated with glutamatergic and GABAergic neurotransmission, including the lateral aspects of the septum and habenula, distinct amygdaloid subnuclei, and the PMH and RMN. DAT-Cre-driven reporter gene expression was evidently not only a result of transcriptional bursting during development as recombinatorial activity remained in adult stages. Based on these results, along with data shown in Allen Institute for Brain Science, we propose that DAT-Cre transgenic mice, which exist in several variants, can be used as transgenic tools for neurocircuitry analysis in Cre-Lox-driven experiments not only for the study of dopaminergic brain systems, but also for non-dopaminergic neurons in limbic neurocircuitry.

## Synthesis

Reviewing Editor: Karen Szumlinski, University of California at Santa Barbara Decisions are customarily a result of the Reviewing Editor and the peer reviewers coming together and discussing their recommendations until a consensus is reached. When revisions are invited, a fact-based synthesis statement explaining their decision and outlining what is needed to prepare a revision will be listed below. The following reviewer(s) agreed to reveal their identity: Madelyn Ray.

In consultation with two expert reviewers, I have reached the decision of Revise-Reviewing Editor Review Only on your manuscript. Overall, we all agreed that the report provides important characterization In this study, the authors provide a thorough anatomical characterization of Cre-driven transgene expression in two different lines of DAT-Cre transgenic mice. Of relevance to the scientific field, significant off-target (i.e., non-dopaminergic) transgene expression was detected. As such, we agree that the data reported in this manuscript is timely and high relevant to any neuroscientist considering employing DAT-Cre mice for their research. This all being said, the reviewers did raise some minor, but important, issues that I would like to see corrected in a revised version of the report. These revisions include:

1. Indication of sample sizes employed.

2. The basis of the DV coordinate for stereotaxic surgery (dura, bregma/surface of the skull etc).

3. Provide citation for the IHC protocol employed

Finally, Reviewer 2 raised additionally concerns. For one, he/she would like the title revised to better reflect the study. It is recommended to change the title to emphasize that the paper is a characterization of ectopic expression in transgene in DAT-Cre mouse lines, given that “multiple opportunities for selective targeting” have yet to be tested directly. Secondly, he/she would like greater discussion regarding the controls for Cre-independent transgene expression. In this report, it is argued that the ectopic reporter expression is Cre-dependent. However, if the transgene with the recombinase sites is not under constant selection, the sites can “leak”, and recombine to express the payload independently of Cre (ex: Prabhakar et al. PLoS One, 2019). Reviewer 2 and I would like to know if the authors have looked at reporter expression in the complete absence of Cre (in the reporter lines non crossed with DAT-Cre mouse?), or after DIO virus injection in wild type mice? If not, then this study limitation should at least be highlighted and discussed in the revised paper.

## Author Response

We thank the Editor and Reviewers for valuable comments, all of which we have addressed. Please see our point-by-point response below. We feel that by responding to these comments and making corrections in the manuscript, that the quality has been improved and we hope it will now be found suitable for publication.

Main comments (1-3):

1. Indication of sample sizes employed.

Response: This information has now been added to the Methods section.

2. The basis of the DV coordinate for stereotaxic surgery (dura, bregma/surface of the skull etc).

Response: Given that we had already provided both sterotaxic coordinates and literature references according to standard nomenclature, we are uncertain of how to

address this comment. The coordinates provided are the ones where the virus was released. Please advise if further information is required.

3. Provide citation for the IHC protocol employed

Response: A full protocol for the immunohistochemistry has now been added to the

Methods section.

Additional comments raised:

“Finally, Reviewer 2 raised additionally concerns. For one, he/she would like the title revised to better reflect the study. It is recommended to change the title to emphasize that the paper is a characterization of ectopic expression in transgene in DAT-Cre mouse lines, given that ”multiple opportunities for selective targeting“ have yet to be tested directly.”

Response:

We agree and have changed the title to:

“Off-target effects in transgenic mice: Characterization of Dopamine transporter (DAT)-Cre transgenic mouse lines exposes multiple non-dopaminergic neuronal clusters available for selective targeting within limbic neurocircuitry”

Given that we provide two examples showing that injection of viral-genetic constructs do indeed validate that the non-dopaminergic expression of Cre recombinase in the lateral habenula and amygdala allows targeting of the floxed eYFP allele, we find that “available for selective targeting” is a less strong than the previous “provides multiple opportinities for selective targeting”, but is well motivated based on the experiments performed. Had these functional experiments not been performed, and our study was only based on in situ hybridization and immunohistochemistry, we would have opted to remove this information from the title.

“Secondly, he/she would like greater discussion regarding the controls for Cre- independent transgene expression. In this report, it is argued that the ectopic reporter

expression is Cre-dependent. However, if the transgene with the recombinase sites is not under constant selection, the sites can ”leak“, and recombine to express the payload independently of Cre (ex: Prabhakar et al. PLoS One, 2019). Reviewer 2 and I would like to know if the authors have looked at reporter expression in the complete absence of Cre (in the reporter lines non crossed with DAT-Cre mouse?), or after DIO virus injection in wild type mice? If not, then this study limitation should at least be highlighted and discussed in the revised paper.”

Response: We agree that this aspect is important and confirm that the control experiments requested have been performed. Firstly, no reporter expression was detected in mice not crossed with DAT-Cre, wildtype mice, or in mice negative for Cre, but positive for the floxed tdTom, i.e tdTom is not expressed in absence of Cre: This information has been added to the Results section.

Further, we have ample experience of using the herein implemented DAT-Cre mice in optogenetics-based approaches after DIO virus injection and have not observed

ectopic reporter gene expression in either DAT-Cre mice injected in the VTA, or indeed in injected wild type mice. Since these control experiments were done in other

context than the current study, this has been addressed in a new section describing this limitation of the study, placed in the Discussion (a reference to previous work by our group is masked to maintain anonymity during the review process).

Additional modifications performed in revised manuscript:

We have updated Table 1-3 as minor inconsistencies were detected.

Previous Supplemental Table is now Table 1.

We also removed the panel showing DAT protein in Table 3 to focus the attention to sites where Dat mRNA was detected (and thus cells that produce

dopamine rather than the ones that are positive for projections).

We have improved the readability of the Figure Legends.

We added a schematic illustration showing the projections identified in the viral-genetic experiment (new Fig 8) to emphasize this finding, which also motivates the title better.

Finally, we have also incorporated a new section in the Results to outline the comparison between our data and that extracted from Allen Brain Atlas in a more readable format with detailed references to Table 3. We believe this addition will improve the clarity of the text and conclusions.
