# Supplementary figures and images for: Off-Target Effects in Transgenic Mice: Characterization of Dopamine Transporter (DAT)-Cre Transgenic Mouse Lines Exposes Multiple Non-Dopaminergic Neuronal Clusters Available for Selective Targeting within Limbic Neurocircuitry
Source: eNeuro. 2019 Oct 8;6(5):ENEURO.0198-19.2019. doi: 10.1523/ENEURO.0198-19.2019 (PMC6873162; doi:10.1523/ENEURO.0198-19.2019)

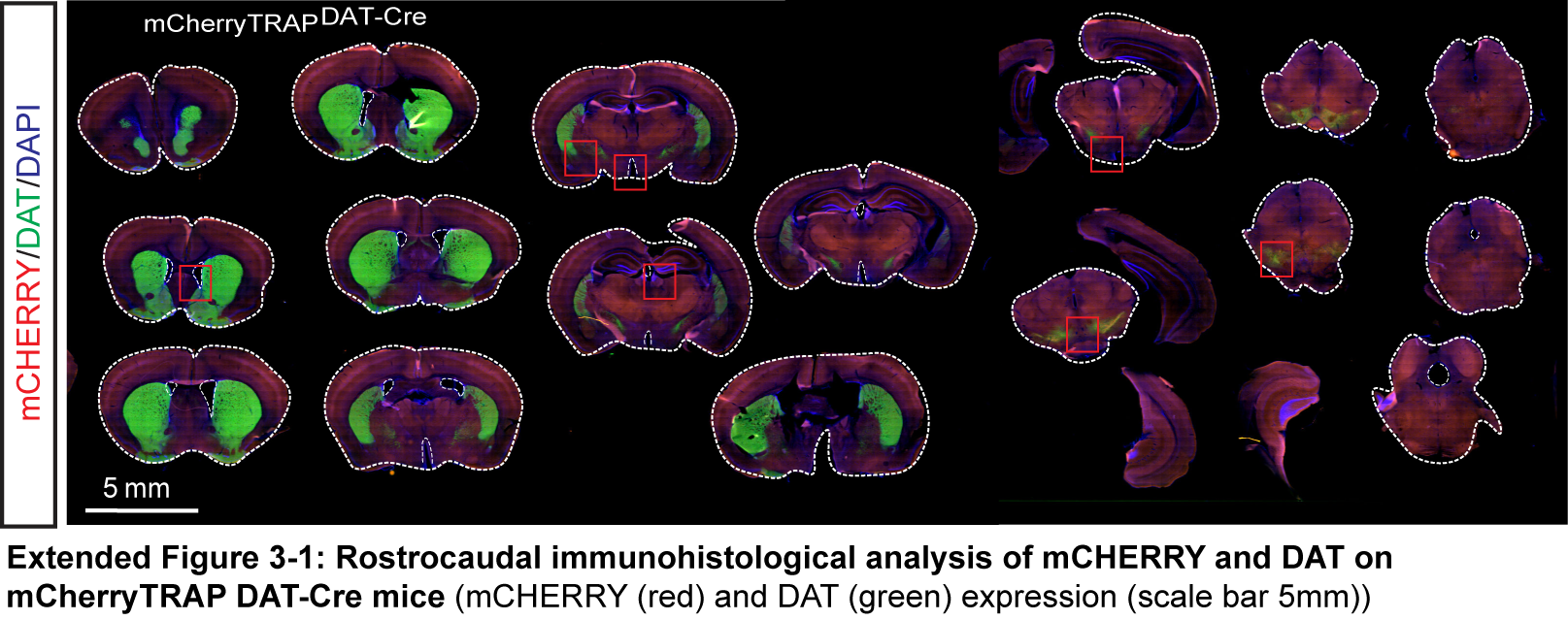

Supplement: Extended Data Figure 3-1 — Rostrocaudal immunohistological analysis of mCHERRY and DAT proteins mCherryTRAP DAT-Cre mice. Immunofluoresence analysis showing detection of mCHERRY (red) and DAT (green) proteins (scale bar: 5 mm) Download Figure 3-1, TIF file. [file sup_enu-eN-NWR-0198-19-s03.tif]

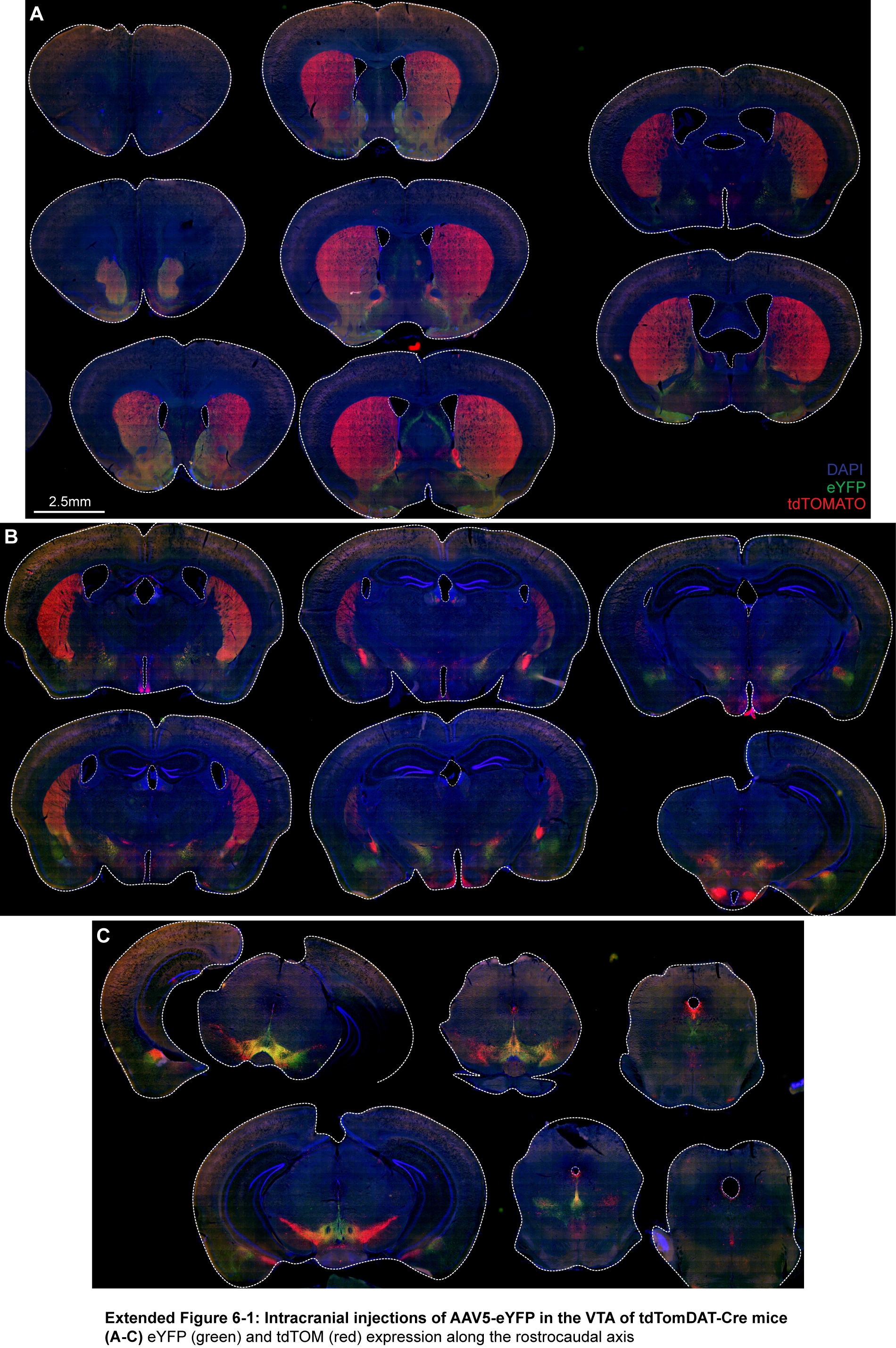

Supplement: Extended Data Figure 6-1 — Intracranial injections of AAV5-eYFP into the VTA of tdTomDAT-Cre mice. A–C, Immunofluorescence analysis of eYFP (green) and tdTOM (red) proteins along the rostrocaudal axis (scale bar: 2.5 mm). Download Figure 6-1, TIF file. [file sup_enu-eN-NWR-0198-19-s04.tif]
